# Public attitudes and practices toward using AI chatbots for healthcare assistance: a multinational cross-sectional study

**DOI:** 10.1186/s12913-025-13832-0

**Published:** 2025-12-30

**Authors:** Aya Elsayed Abdelwahed, Mahmoud Abd El-Nasser, Omar Qasem Heih, Aya Muhammed Suleiman, Ahmed Mithqal Khader, Rahma AbdElfattah Ibrahim, Mohamed Rabiea Abdelnaby Fathalla Hamad, Eslam Radwan, Azza Magdy Srour, HealthTech Alliance, Marwa Mohammed Ibrahim Ghallab

**Affiliations:** 1 Faculty of Medicine, Kafrelsheikh University, Kafr ash Shaykh, Egypt; 2 Faculty of Medicine and Health Sciences, Palestine Polytechnic University, Hebron, Palestine; 3 Internship student, Faculty of Medicine, KafrELShiek University, Kafr ash Shaykh, Egypt; 4 Department of Medical Parasitology, Faculty of Medicine, Kafrelsheikh University, Kafr ash Shaykh, Egypt

**Keywords:** Artificial intelligence, Chatbots, Healthcare, Attitude, Practice, ChatGPT

## Abstract

**Background:**

Using artificial intelligence (AI) chatbots in healthcare can enhance patient care. However, misuse may lead to negative outcomes. Our study’s aim is to evaluate the practices and attitudes related to AI chatbots for healthcare assistance within the general population in the Arab region.

**Methods:**

A population of 12 years old and above from 21 Arab countries was invited to complete a validated web-based questionnaire from 1 May to 1 June 2024. The survey consisted of four sections: demographics, identification, attitudes, and practices related to AI chatbots in healthcare assistance. We utilized Microsoft Excel and SPSS software for data entry and analysis. Descriptive statistics, chi-square tests, and binary logistic regression were used to analyze demographic associations and usage predictors for healthcare

**Results:**

Among the 12,886 valid responses, the median age was 24 years (IQR: 21–31), with a female-to-male ratio of 2:1. Most were single (66.8%), from Egypt (11.2%), urban residents (81.2%), students (43.6%), university-educated (73.2%), or healthcare-affiliated (40.2%). While 72.5% were aware of AI chatbots, only 26.4% used them, primarily for health coaching (67.5%), self-medication (54.5%), self-diagnosis (44.1%), and mental support (48%). ChatGPT was the most used chatbot (22.65%) for healthcare assistance. Individuals with psychological or mental health issues had greater odds of chatbot use (Exp(B) = 1.343, 95% CI: 1.189–1.516, *p* < 0.001), while the strongest predictor was participation in AI-related training courses, which was associated with more than a threefold increase in odds (Exp(B) = 3.109, 95% CI: 2.715–3.559, *p* < 0.001).

**Conclusion:**

This study highlighted varying attitudes and patterns regarding the use of AI-powered chatbots for healthcare assistance, from consultation to self-diagnosis and medication. The insights from this study can help policymakers, researchers, developers and healthcare professionals integrate AI chatbots more effectively into the existing healthcare system.

**Clinical trial number:**

Not applicable.

**Supplementary information:**

The online version contains supplementary material available at 10.1186/s12913-025-13832-0.

## Introduction

Any sufficiently advanced technology is indistinguishable from magic.

       Arthur C. Clarke [1]

Technology plays a vital role in our daily lives [2]. One of the most rapidly evolving technologies is artificial intelligence (AI) powered chatbots. These computer programs use natural language processing (NLP), machine learning (ML), and deep learning (DL) to simulate human conversation [3, 4]. Users can interact with these chatbots through various formats, including text, voice, and images [4].

Since Eliza -the first chatbot- was introduced in 1966, AI chatbots have evolved remarkably [3]. Advanced versions are now widely accessible, including ChatGPT, Siri, Alexa, Gemini, Copilot, and IBM Watson [4]. Factors that contributed to its widespread use include the launch of ChatGPT in 2022 and the integration of these chatbots into social media platforms such as Snapchat, Telegram, and Meta [4–6]. They have become essential to our daily lives, playing significant roles in education, research, business, tourism, and even the healthcare system [7–11].

In healthcare systems, AI chatbots have been shown to assist providers in decision-making related to diagnosis, treatment, health promotion, and patient monitoring [4]. Research highlights the potential for integrating AI chatbots into public healthcare as valuable tools. These chatbots can facilitate health consultations and patient education, offer psychological support, manage appointments, oversee medication adherence, and provide guidance on healthy lifestyle choices [4]. The integration of an AI chatbot in healthcare has the potential to ease the financial burden on both patients and the healthcare system [7]. It can also help reduce energy consumption and lower the carbon footprint associated with healthcare services [4, 12, 13]. AI chatbots are available 24/7, offering patients access to support [14].

Within the Arab region, several national initiatives reflect a growing governmental commitment to digital health transformation [15]. Countries such as the United Arab Emirates and Saudi Arabia have incorporated healthcare-related AI applications into their national AI strategies—such as the UAE Strategy for Artificial Intelligence 2031 and Saudi Arabia’s National Strategy for Data and AI (NSDAI)—which encourage the integration of AI-supported health services [16].

While chatbots can offer several benefits, depending solely on them without consulting human healthcare providers can be detrimental [17]. Errors, hallucinations, and misinformation are major concerns, especially when dealing with complex health conditions that require human expertise for accurate diagnosis and treatment [7]. AI chatbot adoption faces challenges, including algorithmic limitations, regulatory constraints, data reliance, and transparency issues that reduce trust [18]. AI-generated solutions can be flawed, and algorithmic bias presents risks, while data security and privacy remain major concerns. [19]. Limited access to clinical data, time constraints in research, and the complexity of data collection further hinder integration [17, 20]. Recent comparative analyses highlight that healthcare AI has progressed from rule-based expert systems to machine-learning models and now to large language models, revealing persistent gaps in unified evaluation and governance across these technological eras [21].

Evaluating public attitudes and practices regarding the use of AI chatbots for healthcare assistance has become essential. However, research exploring these issues is still limited in the Arab region. Existing studies have focused primarily on healthcare professionals and medical students, who can critically assess the accuracy of chatbot-generated information [22, 23]. In contrast, public attitudes toward AI chatbots in healthcare have not been adequately studied in the Arab region. This raises important questions about their perceived effectiveness in health consultations, cost efficiency, and potential to support or replace human healthcare professionals. Understanding public trust and comfort with AI chatbots is crucial for their effective and healthy integration into healthcare systems [24].

Additionally, the extent and nature of AI chatbot usage for health purposes among the public require further investigation. While some individuals may use chatbots for general health advice, such as lifestyle coaching, others might rely on them for high-risk applications such as self-diagnosis and self-treatment [25, 26]. Distinguishing between specialized medical chatbots and general-purpose models (e.g., ChatGPT) is also essential, as the latter may lack training on high-quality, peer-reviewed medical data, increasing the risk of misinformation [7]. Examining these usage patterns can help improve chatbot training, raise awareness about potential risks, and guide interventions to promote safe and effective use.

Demographic factors and prior AI-related training may influence chatbot adoption for healthcare. Hence, identifying whether specific demographics are strong predictors of chatbot reliance—or whether other factors play a more significant role—can inform future research and policy decisions. Investigating these relationships will provide insights into how AI chatbots can be tailored to meet user needs while minimizing potential risks in healthcare applications.

This study bridges these gaps by assessing public attitudes and practices regarding the use of general chatbots in healthcare assistance in the Arab regions. It also explored the influence of some demographic factors on AI usage in healthcare. The findings provide valuable insights to guide future research and inform policymakers, healthcare providers, and technology developers, supporting effective AI chatbot integration to enhance patient engagement and health outcomes.

## Methods

### Study design and setting

A multinational online descriptive cross-sectional study was implemented among the population of 21 Arab nations (Algeria, Bahrain, Comoros, Egypt, Iraq, Jordan, Kuwait, Lebanon, Libya, Mauritania, Morocco, Oman, Palestine, Qatar, Saudi Arabia, Sudan, Somalia, Syria, Tunisia, the United Arab Emirates, and Yemen).

### Study populations

We included participants who resided in an Arab country, had internet access, and consented to participate in either English or Arabic. Adolescents are active users of social media and AI chatbots, making their inclusion essential for capturing real-world interactions with this technology, particularly in accessing digital health information [27–31]. Hence, we included participants above than 12 years old. The exclusion criteria are those who do not fulfill the inclusion criteria.

### Data collection tool

The questionnaire was developed based on previously established literature [32–34]. It was initially developed in English and then translated into Arabic, and we performed back-translation into English again for verification and accuracy. Content validity was evaluated by six experts, construct validity was assessed using exploratory and confirmatory factor analyses of the scales, face validity was examined through feedback from pilot participants, and reliability was tested using Cronbach’s alpha (see Additional File 1).

The final tool versions (see Additional File 1, Table S1 & S2) consisted of four parts, which are detailed below:

1. ***Demographics -*** This part consists of 10 questions that address age, sex, country, education, residence, occupation, specialties, and history of chronic and mental health diseases.
2. ***Identifying AI Chatbots*** - This part has three questions, in particular:
  - Hearing about AI chatbots.
  - Definition of AI chatbots.
  - Previous training in artificial intelligence.
3. ***Attitudes and confidence in the use of AI chatbots for healthcare assistance:*** This section consists of 13 items with a five-point Likert scale.
4. ***Practice of AI Chatbots in Healthcare Assistance:*** This domain consists of 4 questions. The first question aims to investigate the usage of AI chatbots in fields other than healthcare, with ten possible options. The second question concerns the prevalence of using AI chatbots for healthcare assistance. The participants who answered that they did not use these chatbots for healthcare assistance would submit the form, whereas those who did would transform it into the next question. The third question asks which AI chatbots are used most frequently. The fourth question evaluated the common applications of AI chatbots in healthcare assistance. This question includes 10 options in the Arabic version and only six in the validated English version.

### Sample size and sampling

We calculated the sample size to be 386 respondents from each country via version 7.2.4.0 of the Epi Info software at a confidence level of 95% (α = 0.05) [35]. To account for a 10% nonresponse rate, we aimed to collect at least 423 responses from each country. We utilized a convenience sampling approach for data collection.

### Data collection

We recruited collaborators from each country. They collected data online via Google Forms by sharing questionnaires through various platforms of social media from 1 May to 1 June 2024. The online questionnaire required responses for all applicable items and employed filtering logic to guide completion, resulting in a dataset with no missing values

### Statistical analysis

We organized the data via a Microsoft Excel spreadsheet, which was then imported into the 29th version of the IBM SPSS software to obtain the descriptive statistics for all the variables [36, 37] (see Additional file 1 for item coding).

The only numerical variable was “Age,” so tests for normality were conducted, including the Shapiro test and a boxplot. Both highlighted its skewness and warranted its description in the median (minimum-maximum) form.

For the attitude section, we changed the original 5-point scale to a simpler 3-point scale for easier interpretation (see Additional file 1, Table S3). “Strongly Disagree” and “Disagree” were combined and labeled “Disagree”. The “Neutral” option remains unchanged, whereas “Agree” and “Strongly Agree” are merged into a single category labeled “Agree”.

We conducted chi-square tests with effect size to explore associations between demographic factors and AI chatbot usage for healthcare assistance. To account for multiple comparisons in the Chi-square analyses, we applied the Benjamini-Hochberg False Discovery Rate (FDR) correction for Type I error inflation (see Additional file 1, Table S4). Raw p-values from each test were ranked, and an adjusted significance threshold was calculated using the B-H procedure. Variables with p-values below the adjusted threshold were considered statistically significant. This adjustment ensured a more robust interpretation of the findings while minimizing false-positive results. The comparisons remained significant after correction.

Additionally, binary logistic regression, with the Hosmer–Lemeshow test for model fit, was performed to explore the influence of demographic variables (independent variables), on AI chatbot use (dependent variable). To ensure a meaningful and stable comparison, we selected the reference group as the category with the highest frequency in each variable.

The model fit statistics indicated explanatory power (Nagelkerke R^2^ = 0.106), with a non-significant Hosmer–Lemeshow test (*p* = 0.807), suggesting a good fit.

## Results

### Demographic characteristics

As shown in Tables 1 of the 12911 completed questionnaires, 12886 were included after inconsistent data were removed. The highest percentages of the collected samples were from Egypt (11.2%) and Palestine (10%). The median age of the participants was 24 years old. The female-to-male ratio was 2:1 (65%:33.5%). Nearly two-thirds of the participants were single (66.8%). The majority resided in urban areas (81.2%), had a university education (73.2%), were still students (43.6%), had specialties in the healthcare system (40.2%), and had heard about AI chatbots (72.5%). Almost 14.7% of the participants were diagnosed with a chronic disease, and 18.6% had a mental illness.

**Table 1:** Socio-demographic characteristics of participants

		Frequency (*n* = 12886)	Percent (%)
English version	Arabic	10660	82 × 7
	English	2226	17 × 3
Age (years)	Median (IQR)	24 (21–31)	
Gender	Prefer not to answer	204	1 × 6
	Female	8370	65 × 0
	Male	4312	33 × 5
Marital status	Prefer not to answer	279	2 × 2
	Married	3755	29 × 1
	Single	8609	66 × 8
	Widow	93	0 × 7
	Divorced	150	1 × 2
Residence	Rural	2418	18 × 8
	Urban	10468	81 × 2
Country	Algeria	332	2 × 6
	Bahrain	1051	8 × 2
	Comoros	7	0 × 1
	Djibouti	2	0 × 0
	Egypt	1440	11 × 2
	Iraq	1120	8 × 7
	Jordan	1169	9 × 1
	Kuwait	310	2 × 4
	Lebanon	350	2 × 7
	Libya	810	6 × 3
	Mauritania	5	< 0.01
	Morocco	43	0 × 3
	Oman	994	7 × 7
	Palestine	1295	10 × 0
	Qatar	484	3 × 8
	Saudi Arabia	525	4 × 1
	Somalia	43	0 × 3
	Sudan	659	5 × 1
	Syria	751	5 × 8
	Tunisia	251	1 × 9
	United Arab Emirates	529	4 × 1
	Yemen	716	5 × 6
Education	Non-educated·	141	1 × 1
	Pre-university education	1882	14 × 6
	University education (undergraduate/postgraduate)	9431	73 × 2
	Post-graduate degrees (Master’s/Doctorate) ·	1432	11 × 1
Main occupation	Unemployed	1676	13 × 0
	Governmental employee	2078	16 × 1
	Private employee	1624	12 × 6
	Freelancer	800	6 × 2
	Artisan	87	0 × 7
	Housewife	848	6 × 6
	Student	5614	43 × 6
	Retired	124	1 × 0
	Others	35	0 × 3
Your Specialty (current or future)	Education	1902	14 × 8
	Engineering	1469	11 × 4
	Health care system	5183	40 × 2
	IT/computer science	957	7 × 4
	Business	1183	9 × 2
	Arts/Humanities	661	5 × 1
	Natural science	719	5 × 6
	Other	812	6 × 3
Diagnosed with chronic disease·	Yes	1898	14 × 7
Have any psychological or mental health issues	Yes	2400	18 × 6
Have you heard about AI-powered chatbots (like Chat GPT, Gemini, Poe)	No	3549	27 × 5
	Yes	9337	72 × 5

### Previous training and correct identification

Table 2 shows that 6.7% of the people who heard about AI chatbots identified the correct definition and had previously attended training courses about AI-powered chatbots.

**Table 2:** Identification of AI power chatbots by the public and previous training about AI-powered chatbots·

		I have participated in training courses or workshops about AI-powered chatbots or similar content online· *N* = 9337		Total 100%
		No (*N* = 8170, 87 × 5%)	Yes (*N* = 1167, 12 × 5%)	
Identified AI-powered chatbots as:	Chatbots that are powered by human intelligence·	3562 (38 × 1%)	476 (5 × 1%)	4038 (43 × 2%)
	Chatbots that are programmed to make phone calls·	162 (1 × 7%)	41 (0 × 4%)	203 (2 × 2%)
	Chatbots that rely on natural language processing and machine learning to mimic human conversation	4329 (46 × 4%)	630 (6 × 7%)	4959 (53 × 1%)
	Chatbots that are only used for audio	117 (1 × 3%)	20 (0 × 2%)	137 (1 × 5%)

### Public attitudes and confidence

Regarding the attitude toward using AI chatbots for health care assistance (Table 3), Most agreed that chatbots improve access to health information (54.8%), assist with medication management (45.4%), and support initial symptom assessment (44.1%). A clear majority (46.2%) strongly rejected the idea that chatbots could replace human healthcare professionals.

**Table 3:** The attitude toward using AI chatbots for healthcare assistance

			Frequency (*n* = 9337)	Percent %
I think AI chatbots are effective to:	1. Contribute to health care assistance.	I strongly disagree.	699	7.5
		Disagree.	1400	15.0
		Neutral	2755	29.5
		Agree	3827	41.0
		Strongly agree.	656	7.0
	2.Provide accurate and trustworthy sources of health-related information.	Strongly disagree.	621	6.7
		Disagree.	2011	21.5
		Neutral	3170	34.0
		Agree	3013	32.3
		Strongly agree.	522	5.6
	3. Facilitate the accessibility of health-related information and resources	Strongly disagree.	370	4.0
		Disagree.	725	7.8
		Neutral	1955	20.9
		Agree	5120	54.8
		Strongly agree.	1167	12.5
	4. Be used for identifying the initial symptoms assessment for health-related issues	Strongly disagree.	606	6.5
		Disagree.	1360	14.6
		Neutral	2555	27.4
		Agree	4119	44.1
		Strongly agree.	697	7.5
	5. Assist in medication management and reminders.	Strongly disagree.	500	5.4
		Disagree.	971	10.4
		Neutral	2012	21.5
		Agree	4237	45.4
		Strongly agree.	1617	17.3
	6. Offer psychological and mental support and resources.	Strongly disagree.	814	8.7
		Disagree.	1773	19.0
		Neutral	2993	32.1
		Agree	3145	33.7
		Strongly agree.	612	6.6
	7. Contribute to remote monitoring of health issues	Strongly disagree.	931	10.0
		Disagree.	2021	21.6
		Neutral	2905	31.1
		Agree	2926	31.3
		Strongly agree.	554	5.9
	8. Offer cost-effective solutions for healthcare assistance.	Strongly disagree.	1028	11.0
		Disagree.	2048	21.9
		Neutral	2921	31.3
		Agree	2782	29.8
		Strongly agree.	558	6.0
	9. Replace the human health care professionals.	Strongly agree.	259	2.8
		Agree	942	10.1
		Neutral	1389	14.9
		Disagree.	2429	26.0
		Strongly disagree.	4318	46.2
	10. Help the human health care professionals.	Strongly disagree.	1276	13.7
		Disagree.	1577	16.9
		Neutral	2548	27.3
		Agree	3156	33.8
		Strongly agree.	780	8.4
Regarding using AI chatbots for health care assistance:	1. I feel comfortable when discussing health issues with AI chatbots (or imagining the situation if I haven’t done it before).	Strongly disagree.	1273	13.6
		Disagree.	2330	25.0
		Neutral	3047	32.6
		Agree	2231	23.9
		Strongly agree.	456	4.9
	2. I consider it transparent.	Strongly disagree.	901	9.6
		Disagree.	1989	21.3
		Neutral	3569	38.2
		Agree	2449	26.2
		Strongly agree.	429	4.6
	3. I consider it to be trustworthy.	Strongly disagree.	1451	15.5
		Disagree.	2712	29.0
		Neutral	3245	34.8
		Agree	1616	17.3
		Strongly agree.	313	3.4

### The use of AI chatbots for non-healthcare services

**Fig. 1:**
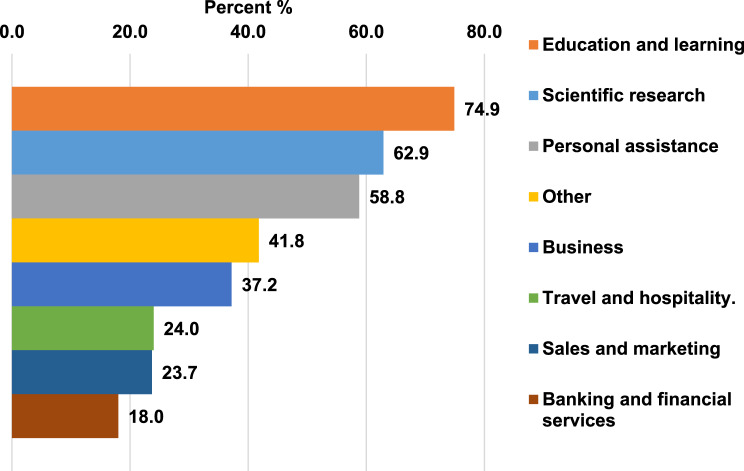
Using AI chatbots for non-healthcare services

The participants reported using AI chatbots for non-healthcare services such as education and learning (74.9%) and scientific research (62.9%) (Fig. 1).

### Prevalence of using AI chatbots for healthcare services

**Fig. 2:**
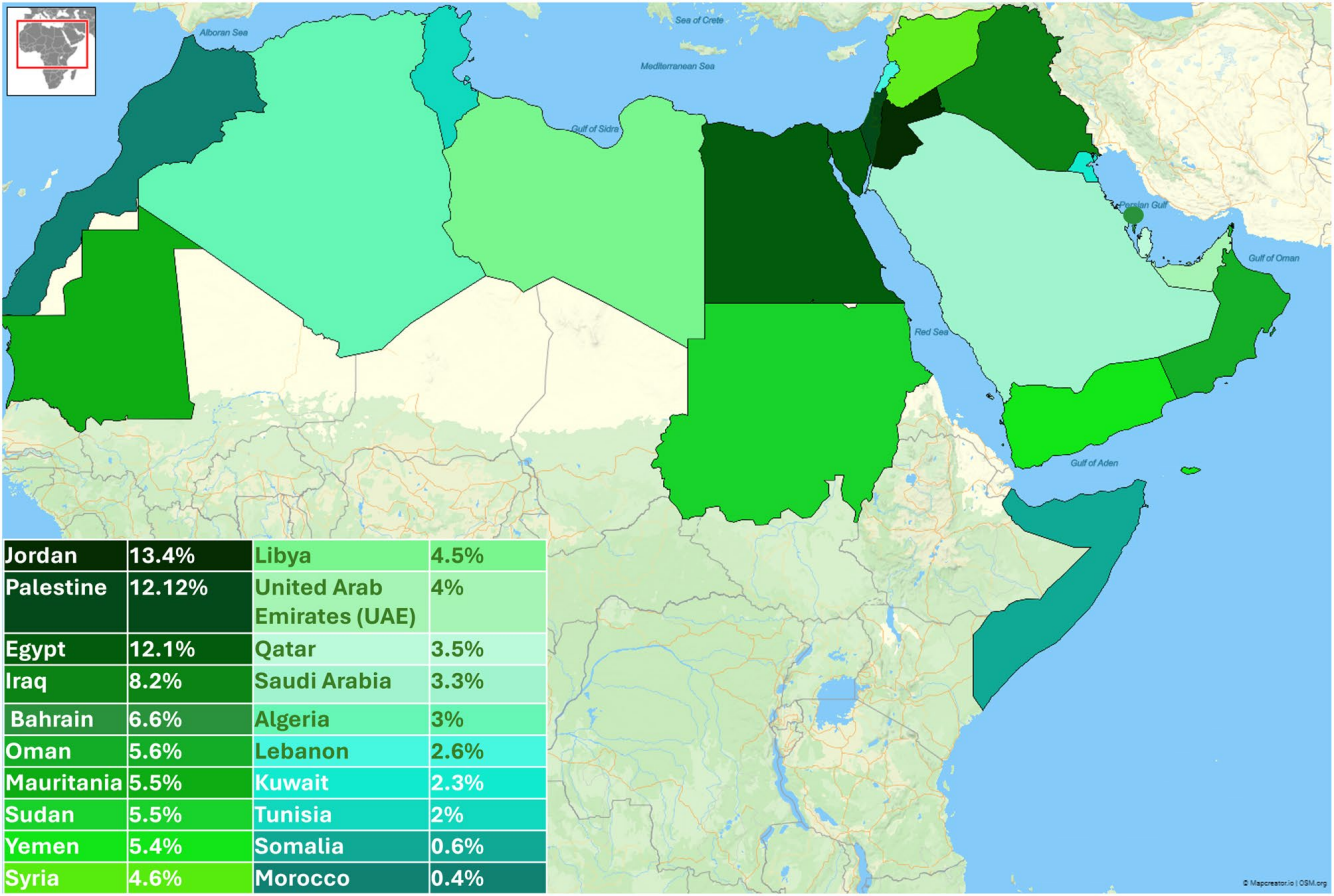
Country-wise prevalence of using AI-powered chatbots for health care assistance

On the other hand, we found that 26.4% of the participants used AI chatbots for health care assistance services. The highest prevalence rates were detected in Jordan (13.4%), Palestine (12.12%), and Egypt (12.1%) (Fig. 2).

**Fig. 3:**
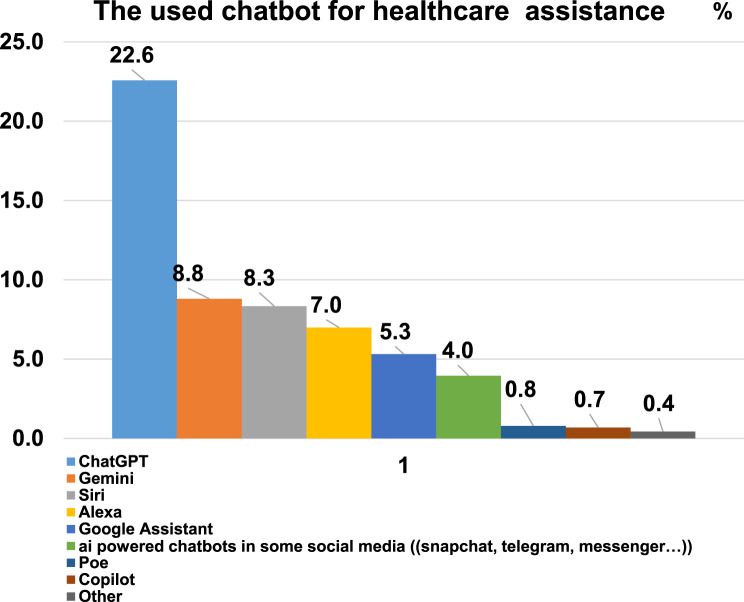
AI-powered chatbots used for healthcare assistance

The common AI chatbots used for healthcare assistance were ChatGPT (22.6%), Google Assistant (8.8%), and Siri (8.3%) (Fig. 3).

### The purpose of using AI chatbots for healthcare services

As presented in Table 4, the commonest uses of AI chatbots for health care assistance were offering personalized health coaching (67.5%), identifying the initial symptoms of the disease (58.2%), and obtaining information about self-medication (54.5%).

**Table 4:** Using AI chatbots for health care assistance

	Frequency (*N* = 2461)	Percent %
1. Medication management (and/or) Reminders	920	37.4
2. Assisting with appointment scheduling and reminders related to health (such as check-ups, medications, and any health regimen)	884	35.9
3. Facilitating online health consultations	1307	53.1
4. Getting information about self-medication (Taking medicines without physician consultation)	1342	54.5
5. Offering personalized health coaching (such as promoting a healthy lifestyle with exercising, a healthy diet, quitting smoking, and more.)	1660	67.5
6. Online nursing and monitoring services	778	31.6
7. Identifying the initial symptoms of the disease	1090	58.2
8. Self-diagnosis	826	44.1
9. Psychological and Mental support	899	48
10. Others	1014	54.2

### The association between demographics and the use of AI chatbots for healthcare

The chi-square test (Table 5) reveals significant but weak differences of using of AI chatbots for healthcare assistance in the following factors: age (*p* < 0.001, Cramer’s V = 0.094), gender (*p* < 0.001, Cramer’s V = 0.076), marital status (*p* < 0.001, Cramer’s V = 0.071), education level (*p* < 0.001, Cramer’s V = 0.050), and employment status (*p* < 0.001, Cramer’s V = 0.079). Additionally, field of specialty (*p* < 0.001, Cramer’s V = 0.135), mental health status (*p* < 0.001, Cramer’s V = 0.055), and prior AI training (*p* < 0.001, Cramer’s V = 0.169) were also significant factors, with prior AI training showing the strongest association, although it remains weak.

**Table 5:** Chi-square test between using AI chatbots for healthcare assistance and demographic characteristics

Chi-square test for using AI chatbots for healthcare assistance						
		Have you ever used AI chatbots for healthcare assistance?				
		Yes (*n* = 2461%))	χ^2^ Value	df	Cramer’s V	p-value
Age categories	< 16	12 (0.5%)	82.027	6	0.094	** < 0.001**
	16–24	1633 (66.4%)				
	25–34	548 (22.3%)				
	35–44	165 (6.7%)				
	45–54	72 (2.9%)				
	55–64	24 (1%)				
	> 64	7 (0.3%)				
Gender	Prefer not to answer	28 (1.1%)	53.434	2	0.076	** < 0.001**
	Female	1420 (57.7%)				
	Male	1013 (41.2%)				
Marital Status	Prefer not to answer	35 (1.4%)	47.671	4	0.071	** < 0.001**
	Married	454 (18.4%)				
	Single	1948 (79.2%)				
	Widow	6 (0.2%)				
	Divorced	18 (0.7%)				
Residence	Rural	434 (17.6%)	0.965	1	0,010	0.326
	Urban	2027 (82.4)				
Education	Non-educated.	14 (0.6%)	23.141	3	0.050	** < 0.001**
	Pre-university education	228 (9.3%)				
	University education (undergraduate/postgraduate)	1944 (79%)				
	Post-graduate degrees (Master’s/Doctorate).	275 (11.2%)				
Main occupation	Unemployed	304 (12.4%)	57.745	8	0.079	** < 0.001**
	Governmental employee	276 (11.2%)				
	Private employee	291 (11.8%)				
	Freelancer	146 (5.9%)				
	Artisan	10 (0.4%)				
	Housewife	68 (2.8%)				
	Student	1350 (54.9%)				
	Retired	7 (0.3%)				
	Others	9 (0.4%)				
Your Specialty (current or future)	Education	244 (9.9%)	170.312	7	0.135	** < 0.001**
	Engineering	249 (10.1%)				
	Health care system	1344 (54.6%)				
	IT/computer science	199 (8.1%)				
	Business	162 (6.6%)				
	Arts/Humanities	82 (3.3%)				
	Natural science	119 (4.8%)				
	Other	62 (2.5%)				
Diagnosed with chronic disease.	No	2123 (86.3%)	0.247	1	0.005	0.619
	Yes	338 (13.7%)				
“Do you have any psychological or mental health issues?”	No	1890 (76.8%)	27.874	1	0.055	** < 0.001**
	Yes	571 (23.2%)				
I have participated in training courses or workshops about AI-powered chatbots or similar content online.	No	1923 (78.1%)	267.841	1	0.169	** < 0.001**
	Yes	538 (21.9%)				

χ^2^ Value – Chi-square Value. df – Degrees of Freedom. The table shows weak but significant associations between AI chatbot use and age, specialty, gender, mental health, and AI training, while residence and chronic disease have no impact

As shown in Table 6, logistic regression analysis revealed several significant predictors of AI chatbot usage for healthcare assistance. Compared with those with university education, individuals with no education (*p* = 0.001, Exp(B) = 0.222, 95% CI: 0.090–0.547), pre-university education (*p* = 0.001, Exp(B) = 0.230, 95% CI: 0.094–0.560), and postgraduate degrees (*p* = 0.006, Exp(B) = 0.281, 95% CI: 0.114–0.694) were significantly less likely to use AI chatbots.

**Table 6:** Binary logistic regression test between using AI chatbots for healthcare assistance and demographic predictors

Have you ever used AI chatbots for healthcare assistance (yes/No)								
*N* = 9337	B	S.E.	Wald	Df	Sig.	Exp(B)	95% C.I.for EXP(B)	
							Lower	Upper
**Age categories (Reference: 16–24)**			21.727	6	**0.001**			
< 16	0.572	0.341	2.807	1	0.094	1.771	0.907	3.457
25–34	0.419	0.350	1.435	1	0.231	1.521	0.766	3.021
35–44	0.179	0.365	0.239	1	0.625	1.196	0.584	2.445
45–54	−0.086	0.380	0.051	1	0.821	0.918	0.436	1.933
55–64	−0.029	0.433	0.005	1	0.946	0.971	0.416	2.269
> 64	0.933	0.609	2.347	1	0.125	2.542	0.771	8.383
**Gender (Reference: Female)**			60.758	2	** < 0.001**			
Prefer not to answer	0.028	0.233	0.014	1	0.905	1.028	0.652	1.623
Male	0.451	0.234	3.696	1	0.055	1.569	0.991	2.485
Marital Status (Reference: Single)			1.296	4	0.862			
Prefer not to answer	0.168	0.224	0.562	1	0.453	1.183	0.763	1.835
Married	0.132	0.211	0.392	1	0.531	1.141	0.755	1.724
Widow	−0.143	0.540	0.070	1	0.791	0.867	0.301	2.499
Divorced	−0.023	0.352	0.004	1	0.947	0.977	0.490	1.948
**Country (Reference: Egypt)**			83.254	20	** < 0.001**			
Algeria	0.478	0.174	7.521	1	**0.006**	1.612	1.146	2.268
Bahrain	1.173	1.077	1.186	1	0.276	3.231	0.391	26.675
Comoros	0.069	0.158	0.194	1	0.660	1.072	0.787	1.460
Iraq	0.240	0.165	2.103	1	0.147	1.271	0.919	1.758
Jordan	0.568	0.159	12.724	1	** < 0.001**	1.764	1.292	2.411
Kuwait	0.380	0.220	2.990	1	0.084	1.462	0.951	2.248
Lebanon	−0.001	0.207	0.000	1	0.997	0.999	0.666	1.499
Libya	0.011	0.181	0.004	1	0.953	1.011	0.709	1.441
Morocco	0.322	0.414	0.603	1	0.437	1.380	0.612	3.108
Oman	0.037	0.176	0.045	1	0.833	1.038	0.736	1.464
Palestine	0.311	0.160	3.769	1	0.052	1.364	0.997	1.867
Qatar	−0.043	0.190	0.050	1	0.822	0.958	0.661	1.390
Saudi Arabia	0.153	0.195	0.618	1	0.432	1.166	0.795	1.709
Somalia	1.514	0.452	11.218	1	**0.001**	4.543	1.874	11.014
Sudan	0.095	0.176	0.289	1	0.591	1.099	0.778	1.553
Syria	−0.173	0.177	0.951	1	0.329	0.841	0.594	1.191
Tunisia	−0.088	0.221	0.160	1	0.689	0.916	0.594	1.411
United Arab Emirates	0.290	0.189	2.346	1	0.126	1.336	0.922	1.937
Yemen	0.445	0.179	6.168	1	**0.013**	1.560	1.098	2.215
**Residence (Reference: Urban) Rural**	−0.056	0.068	0.672	1	0.413	0.946	0.828	1.080
**Education (Reference: University education (undergraduate/postgraduate))**			15.657	3	**0.001**			
Non-educated.	−1.504	0.460	10.708	1	**0.001**	0.222	0.090	0.547
Pre-university education	−1.470	0.455	10.457	1	**0.001**	0.230	0.094	0.560
Post-graduate degrees (Master’s/Doctorate).	−1.268	0.461	7.567	1	**0.006**	0.281	0.114	0.694
**Main occupation (Reference: Student)**			13.981	8	0.082			
Unemployed	−0.210	0.114	3.398	1	0.065	0.811	0.649	1.013
Government employee	−0.046	0.107	0.182	1	0.670	0.955	0.774	1.179
Private employee	0.139	0.128	1.191	1	0.275	1.149	0.895	1.476
Freelancer	−0.216	0.408	0.281	1	0.596	0.805	0.362	1.793
Artisan	0.224	0.173	1.681	1	0.195	1.251	0.892	1.754
Housewife	0.027	0.081	0.111	1	0.739	1.027	0.876	1.205
Retired	−0.434	0.434	0.999	1	0.318	0.648	0.277	1.517
Others	0.463	0.426	1.180	1	0.277	1.589	0.689	3.664
**Your Specialty (current or future) (Reference: Health care system)**			152.093	7	** < 0.001**			
Education	−0.263	0.110	5.751	1	**0.016**	0.769	0.620	0.953
Engineering	0.462	0.089	27.109	1	** < 0.001**	1.587	1.334	1.888
IT/computer science	−0.202	0.118	2.934	1	0.087	0.817	0.648	1.030
Business	−0.187	0.122	2.362	1	0.124	0.829	0.653	1.053
Arts/Humanities	−0.295	0.148	3.972	1	**0.046**	0.744	0.557	0.995
Natural science	−0.062	0.134	0.211	1	0.646	0.940	0.723	1.223
Other	−0.540	0.161	11.177	1	**0.001**	0.583	0.425	0.800
**Diagnosed with chronic disease. (Reference: No)**	0.106	0.075	1.978	1	0.160	1.112	0.959	1.288
**Have psychological or mental health issues (Reference: No)**	0.295	0.062	22.515	1	** < 0.001**	1.343	1.189	1.516
**I have participated in training courses or workshops about AI-powered chatbots or similar online content (Reference: No)**	1.134	0.069	269.557	1	** < 0.001**	3.109	2.715	3.559

B – Beta coefficient. S.E. – Standard Error. df – Degrees of Freedom. Sig. – Significance level. Exp(B) – Exponentiated Beta coefficient. 95% C.I. for Exp(B) − 95% Confidence Interval for Exp(B). Lower – Lower bound of the confidence interval. Upper – Upper bound of the confidence interval. AI chatbot use is weakly predicted by age, gender, education, specialty, mental health, and AI training, with training being the strongest predictor. Model fit: Nagelkerke R^2^ = 0.106, Hosmer-Lemeshow χ^2^ = 4.525, *p* = 0.807 (good fit)

Regarding specialty, individuals in engineering (*p* < 0.001, Exp(B) = 1.587, 95% CI: 1.334–1.888) were more likely to use AI chatbots than those in healthcare. Conversely, those in education (*p* = 0.016, Exp(B) = 0.769, 95% CI: 0.620–0.953), arts/humanities (*p* = 0.046, Exp(B) = 0.744, 95% CI: 0.557–0.995), and other specialties (*p* = 0.001, Exp(B) = 0.583, 95% CI: 0.425–0.800) were significantly less likely to use AI chatbots.

Having psychological or mental health issues was a significant predictor, with affected individuals being more likely to use AI chatbots (*p* < 0.001, Exp(B) = 1.343, 95% CI: 1.189–1.516). Finally, participation in AI-related training courses or workshops was the strongest predictor, significantly increasing the likelihood of chatbot usage (*p* < 0.001, Exp(B) = 3.109, 95% CI: 2.715–3.559).

## Discussion

In the digital age, remarkable technological innovations have transformed human-computer interactions. An impressive advancement is the emergence of AI-powered chatbots, particularly after ChatGPT launched in 2022 [5, 6]. Previous studies have shown the impact of AI chatbots on various daily activities, including planning, time management, task completion, education, psychological support, and research [38–40]. However, there is a lack of research focusing on the public’s use of AI chatbots for healthcare assistance. Implementing these chatbots in healthcare without the involvement of healthcare providers may result in malpractice. Therefore, this study aims to evaluate the use of AI chatbots for healthcare assistance by the public in the Arab region.

Our study revealed that more than two-thirds (72.5%) of the participants were familiar with AI-powered chatbots. This finding is consistent with those of previous studies, which reported familiarity rates ranging from 60.5% among medical researchers to 78.6% among Lebanese university students [39, 41–43]. Almost half of our respondents (53.1%) correctly identified AI chatbots, only 6.7% had previously received training about them, and 46.4% had not. This aligns with earlier research regarding acceptable knowledge and familiarity with AI chatbots, even though most participants lacked prior training [39, 43, 44]. One possible explanation for the widespread awareness of AI chatbots without prior training may be their user-friendly interfaces, which simplify technology and enhance user engagement [45, 46]. Another leading factor may be the reach of social media [47]. Users frequently share their experiences with AI chatbots on various platforms, which helps disseminate information about them [47]. Additionally, AI chatbots have been integrated into several social media platforms, such as Snapchat, increasing exposure to this technology for 383 million daily users [45].

We found that 26.4% of the participants reported using AI chatbots for healthcare assistance in the Arab region. The prevalence varies by country, with the highest rates observed in Jordan (13.4%), Palestine (12.2%), and Egypt (12.1%). Conversely, the lowest rates are observed in Morocco (0.4%), Somalia (0.6%), Tunisia (2%), and Kuwait (2.3%). An earlier study revealed that 18.4% of Saudi healthcare workers used AI-powered chatbots to deliver health assistance [48]. While we found that Saudi Arabia had a 3.3% prevalence rate of using AI chatbots for healthcare assistance, this rate was derived from a broader population that included the public, not just healthcare workers. The variations in prevalence rates may be explained by national factors affecting digital health adoption, such as government policies, healthcare systems, internet access, economic conditions, and public trust in technology [49–52].

Nearly two-thirds of the participants (67.5%) reported using AI chatbots for health assistance to receive personalized health coaching that promotes a healthy lifestyle through exercise, diet, and the cessation of bad habits. This finding aligns with previous systematic reviews highlighting similar studies that used AI chatbots for effective behavioral changes and healthy lifestyle modification, which included smoking cessation, medication and diet adherence, exercise compliance, and reducing substance misuse [53, 54]. However, AI chatbots rely on user input, which may be inaccurate, leading to misleading recommendations [7].

Almost one-third of the respondents utilized these chatbots for medication management (37.4%), appointment scheduling and reminders related to health events (35.9%), and online nursing and monitoring services (31.6%). Previous studies have also shown promising results for chatbots used in these areas [17]. Specifically, the study noted a 47% increase in appointments booked via AI chatbots [55]. Additionally, other studies reported that chatbots used for online nursing and monitoring services achieved a satisfaction rate of approximately 85% in terms of quality [56, 57]. We can explain these findings by highlighting that AI chatbots provide 24/7 availability, reduce wait times, and streamline scheduling [14]. Online nursing offers instant responses and remote monitoring, enhancing patient support without the need for in-person visits [14]. For medication management, chatbots help with reminders, dosage tracking, and adherence monitoring, decreasing the risk of missed doses [58]. This may guide future efforts to enhance these tasks through continuous training and improvement. Regular evaluations of user satisfaction and performance are crucial for ensuring effectiveness, addressing technical issues, and optimizing healthcare implementation.

Approximately half of the participants employed AI chatbots to facilitate online health consultations (53.1%), provide mental health support (48%), and identify initial symptoms of diseases (58.2%). Relying on AI chatbots carries risks of misdiagnosis, misinformation, and a lack of personalized care, emphasizing the need for human oversight [59]. To mitigate these risks, users should verify AI-generated advice with healthcare professionals, use chatbots as a supplementary tool rather than a primary source, and ensure the chatbot is from a reputable, medically validated source. These patterns further highlight the ethical challenges emphasized in recent reviews, which underscore the critical importance of safeguarding privacy, preventing bias, ensuring transparency, and maintaining human oversight when AI tools are used for mental health support [60]. in this context, it is important to recognize that many general-purpose chatbots are trained predominantly on western datasets, which may limit cultural sensitivity and introduce biased or culturally inappropriate responses for Arab users. Additionally, given the sensitive nature of mental health information, users may not be fully aware of how their data is collected, stored, or shared, raising significant privacy and confidentiality concerns [61]. Our findings are consistent with reports from the National Health Service (NHS). It is noted that approximately 1.2 million people used chatbots for consultations instead of calling the NHS for nonemergency issues [57]. Various studies have also documented the use of AI chatbots to address mental health conditions and identify initial symptoms [57]. Some studies have addressed the fear of developing an emotional relationship with AI-powered chatbots after they receive some form of mental support [62–64]. This observation could suggest a direction for future research regarding this phenomenon.

Surprisingly, 54.5% of the participants reported using AI chatbots to obtain information about self-medication, whereas 44.1% used them for self-diagnosis. These findings correspond with previous research indicating that 78.4% of the participants intended to use chatbots for self-diagnosis and treatment [32]. The results may reflect a growing dependence on these tools for personal health decisions. The accessibility and cost-effectiveness of these chatbots are likely contributing factors to their popularity [7]. However, relying solely on AI chatbots is potentially dangerous. AI chatbots rely on large datasets and pattern recognition rather than deep clinical reasoning [7, 65]. They are trained in general health information and may not account for individual patient comorbidities or nuanced symptom presentations. Because they lack real-time access to a patient’s full medical context and cannot perform physical examinations or diagnostic tests, they may oversimplify symptoms or miss serious underlying conditions [7, 65, 66]. Additionally, their recommendations are often based on statistical likelihoods and may not reflect the latest clinical guidelines or be adapted to specific patient needs [7]. This can lead to delayed diagnoses, inappropriate use of medications, adverse drug interactions, and even the masking of critical warning signs. Moreover, users may develop a false sense of security, opting to skip necessary medical consultations. Therefore, while chatbots can serve as initial sources of information, they should not replace professional medical evaluation and guidance.

Research has shown that the public tends to trust AI chatbots for health-related information more when individuals are experiencing mild disease symptoms than when they are experiencing severe symptoms [67]. This may be due to the lower perceived risk in mild cases, whereas severe symptoms prompt a preference for human experts for reassurance. However, Meyrowitsch DW and colleagues discussed the dangers of health misinformation caused by algorithms used in public AI chatbots [59]. Therefore, it is advisable not to rely solely on these chatbots for health-related information, particularly for self-diagnosis and medication.

In our study, we found that the most used AI chatbots for healthcare assistance are ChatGPT (22.6%), Google Assistant (8.8%), Siri (8.3%), and AI-powered chatbots on social media platforms such as Snapchat (7%). These findings align with those of previous studies, indicating that ChatGPT is among the most frequently utilized AI chatbots across various populations [48, 68, 69]. Furthermore, the availability of Siri and Google Assistant on mobile devices contributes to their higher usage. Similarly, integrating AI chatbots into social media platforms with large user bases encourages more people to use these technologies. The user-friendly interfaces of these chatbots have made it easier for individuals to understand and engage with technology [45, 46]. Additionally, continuous improvements and updates have helped familiarize the public with these tools and ensure the delivery of high-quality services [70, 71]. These factors likely contribute to building and gaining people’s trust in using these technologies for healthcare assistance. General AI chatbots, like ChatGPT, can offer general health information but may not provide the precise, evidence-based advice needed for personal healthcare [20, 72]. They are designed to provide broad, conversational information across a wide range of topics, including health. While they can offer useful general knowledge, symptom explanations, or lifestyle tips, they are not equipped to deliver precise, evidence-based medical advice tailored to an individual’s unique health profile [7]. These chatbots lack access to a patient’s full medical history, current medications, diagnostic test results, and other critical contextual information necessary for accurate clinical decision-making. As a result, they may unintentionally provide incomplete, outdated, or nonspecific guidance [7]. Therefore, users should take practical steps when using these tools for health issues. These include checking the information with qualified healthcare professionals, comparing it with established clinical guidelines, and ensuring that personal data is handled securely. In contrast, specialized medical chatbots are designed specifically for healthcare. They use carefully selected medical data and follow strict privacy and regulatory rules, making them more reliable and accurate for personalized medical advice [73].

Regarding attitudes toward using AI-powered chatbots for healthcare assistance, 37.9% of the participants agreed that AI chatbots could offer accurate and trustworthy sources of health-related information, and 67.3% believed that AI chatbots could increase the accessibility of health-related resources. Moreover, 48% of the respondents considered AI chatbots to be transparent. These findings highlight the public’s confidence in using AI chatbots for health-related issues. These attitudes may explain the use of chatbots for self-medication information, diagnosis, and personalized health coaching.

Conversely, 38.6% of the participants disagreed that they felt comfortable when using AI chatbots for health issues. Low levels of trust and comfort may limit user engagement, decrease adherence to chatbot recommendations, and undermine their effectiveness as tools for health communication during implementation. As a result, chatbots risk being underutilized or misused, especially in sensitive or complex health scenarios. Several factors may contribute to people’s discomfort when they use AI chatbots. Transitioning from human health professionals to AI chatbots for health-related problems can be unsettling. Individuals may have concerns about technological limitations and issues related to trust (28.2%) and transparency (22.5%), which have been reported. To address these challenges, efforts must focus on improving transparency, aligning chatbot responses with evidence-based guidelines, ensuring data security, and integrating chatbots into healthcare settings with oversight from professionals to build user confidence and promote safe adoption.

Many respondents acknowledged the importance of AI chatbots in contributing to healthcare assistance (48%). They agreed on the effectiveness of using them to assess the initial symptoms of health-related issues (51.6%), and in medication management and reminders (62.7%). This aligns with a study of NHS health professionals, which revealed that 79% deemed the importance and effectiveness of using AI chatbots for healthcare assistance [74]. Another cross-sectional study detected the usefulness and satisfaction of AI-assisted symptom checkers and various forms of healthcare assistance [75]. Using chatbots among healthcare professionals is promising since they can filter true and false information; however, the public should not rely on chatbots solely to avoid potentially harmful consequences in real-time.

Over one-third of the participants agreed that AI chatbots could contribute to the remote monitoring of health-related issues (37.3%) and provide cost-effective solutions for healthcare assistance (35.8%). This may indicate a growing acceptance of digital health, especially following the COVID-19 pandemic, during which we relied on virtual consultations and remote monitoring [76, 77]. In terms of cost-effectiveness, this trend may be attributed to rising healthcare costs and limited resources [78]. Telemonitoring can help reduce the financial burden of traditional healthcare delivery, particularly for mild cases. These findings are consistent with previous research, which has predicted the promising effectiveness of using chatbots as part of telemedicine for health monitoring [79].

The majority of participants (72.3%) did not believe that AI chatbots can replace human healthcare professionals. However, nearly 13% agree that AI chatbots could take place, which is concerning. Relying solely on AI chatbots without consulting human doctors could lead to harmful health consequences in real-time [80, 81]. On the other hand, 42.2% of the participants agreed that chatbots are helpful tools for healthcare professionals in delivering effective healthcare. These findings align with previous studies highlighted the preference for integrating human doctors into the diagnosis and treatment process rather than replacing them [34, 80, 82, 83].

Our study revealed that 40.2% of the participants believed that AI chatbots could effectively provide psychological and mental support. There is a growing body of research highlighting the benefits of AI-powered chatbots in mental health care [84, 85]. However, a study of 971 psychiatrists in 22 countries revealed that 83% of them doubted that AI chatbots could ever deliver empathetic care comparable to that of a psychiatrist [86].

In our study, age weakly influenced chatbot adoption, with younger individuals being more likely to use digital tools. However, age alone was not a strong predictor, as differences among older age groups were minimal when considering other factors. This suggests that while younger individuals may be more technologically inclined, age alone does not fully explain chatbot adoption. Our findings supported previous studies indicating that age is a weak factor in digital health and technology adoption [87, 88]. Enhancing digital literacy, offering socioeconomic support, and promoting personalized engagement may be more effective strategies for increasing AI chatbot usage beyond the age factor [87–89].

We noticed that education was significantly linked to AI chatbot usage (*p* < 0.001). Individuals with no education, pre-university education, or even postgraduate degrees were less likely to use chatbots for health assistance than those with university education. This trend may stem from differences in digital literacy, technological access, and perceived relevance [90, 91]. University graduates often have greater exposure to digital tools, while postgraduates may rely on specialized resources instead [92]. Meanwhile, those with lower education levels may encounter accessibility barriers [91]. Additionally, confidence in technology and socioeconomic factors may play crucial roles, highlighting the need for digital training beyond formal education. Notably in our study, the strongest predictor of AI chatbot use was participation in AI training which significantly increased usage, emphasizing the importance of exposure and hands-on engagement in driving technological adoption.

Professionals in engineering fields showed significantly greater usage of AI chatbots compared to health care professionals, likely because of their frequent interaction with digital tools and AI-driven systems, making them more comfortable with chatbot use [68]. In contrast, individuals in education, arts, and humanities fields demonstrated lower usage compared to heath care filed. This is consistent with previous studies showing that engineering students tend to use AI more than those in medical or humanities disciplines do [68].

Interestingly, chronic disease history was not significantly associated with chatbot usage, whereas individuals with psychological or mental health issues were more likely to use chatbots. Chronic disease patients hesitate to use chatbots, preferring human interaction to manage their condition, despite studies indicating a promising acceptance rate for future use [93, 94]. In contrast, chatbots are more accessible for mental health support, as they rely on conversation-based interactions, which may be sufficient for various forms of mental support [95]. Individuals with mental health concerns may favor chatbots for their privacy and non-judgmental nature, which helps reduce the stigma of seeking professional help, a particularly significant factor in Arab countries [96, 97]. Despite the promising potential of chatbots for mental health support, relying solely on them may have negative consequences due to accuracy concerns [95]. We encourage raising awareness about the risks of misuse and the importance of human consultation for proper mental health care.

Importantly, developing and implementing algorithms and devices that ensure high quality and accuracy in data analysis takes time. Healthcare practitioners and medical AI developers must collaborate to improve this process. Additionally, developers should provide doctors with more information about the models, algorithms, and data that AI chatbots utilize. This transparency can help build confidence in AI conclusions and address potential concerns regarding its use in mental health care.

### Strengths and limitations

This multinational study is addressing a gap in attitudes and practices regarding the use of AI chatbots for healthcare assistance among the public in the Arab region. It includes diverse populations, various demographics, and a large sample size from over 21 countries. We believe that this study can offer valuable insights that can assist researchers, policymakers, healthcare providers, and technology developers in formulating effective strategies for the integration of AI chatbots into healthcare systems, thereby enhancing patient engagement and health outcomes.

This study has some limitations that should be acknowledged. First, a cross-sectional design inherently restricts our ability to infer causality, limiting interpretations to associative relationships. Second, the use of a convenience sampling approach significantly limits the generalizability of the findings. Participants were selected based on accessibility, which introduces selection and sampling biases. This is reflected in the overrepresentation of healthcare-affiliated respondents (40.2%) and university-educated individuals (73.2%), potentially skewing the data toward more informed or technologically engaged perspectives. Third, although data were collected from 21 countries, countries like Egypt contributed disproportionately to the sample (11.2%), while others, such as Djibouti and Mauritania, were minimally represented ( < 0.1%). As such, meaningful cross-country comparison is limited, and caution should be exercised when generalizing findings to broader populations or specific national contexts.

Additionally, the online-only survey format may have excluded individuals without reliable internet access or those with limited digital literacy—an important consideration given the study’s focus on digital health technologies like AI chatbots. The reliance on self-reported responses may introduce recall bias or social desirability bias. The chi-square tests revealed statistically significant but weak associations (Cramer’s V ranging from 0.050–0.169), indicating small effect sizes that may limit the practical significance of these findings. Finally, the binary logistic regression model’s low explanatory power suggests that unmeasured variables may play a significant role in influencing chatbot adoption.

### Recommendations

Future research should explore additional factors influencing AI chatbot adoption for healthcare via longitudinal studies and qualitative interviews. These factors may include income levels, digital literacy, internet accessibility, and specific health conditions such as chronic diseases or mental health conditions. Future studies should distinguish between general-purpose AI chatbots and medically specialized, validated health chatbots to more accurately evaluate their respective roles, risks, and utility. Conducting more targeted, country-level analyses or comparative studies will help generate deeper, context-sensitive insights into how cultural diversity influences public attitudes and practices toward AI-based healthcare tools.

Policymakers and healthcare professionals must raise awareness about the limitations of relying solely on chatbots without human consultation. Enhancing digital literacy through targeted training campaigns can improve adoption, while developers and stakeholders should focus on refining AI models to address public concerns and ensure effective integration into healthcare systems.

## Conclusion

The majority of the adult population in Arab countries involved in healthcare has heard about AI chatbots. Most participants recognized their capabilities, although few had received prior training in them. Regarding their attitudes towards AI chatbots, participants agreed that these tools are effective in facilitating access to health-related information and resources, as well as assisting with medication management and reminders.

However, there were concerns about the accuracy, transparency, and trustworthiness of the health-related information provided by AI chatbots. Many participants rejected the idea that AI could replace human healthcare professionals. Most reported using AI chatbots in nonhealthcare sectors, particularly in education, scientific research, and personal assistance, with a preference for ChatGPT over other chatbot options.

In healthcare contexts, participants indicated that they use AI chatbots as assistants for personalized health coaching, identifying initial symptoms of diseases, and managing self-medication. However, they were less inclined to use them for online nursing and monitoring services. AI chatbot adoption is significantly influenced by education level, field of specialty, and AI-related training, highlighting the importance of digital literacy and exposure in driving usage. Our findings highlight the need for targeted education and further research for the effective integration of AI chatbots for healthcare assistance.

## Electronic supplementary material

Below is the link to the electronic supplementary material.

Supplementary Material 1

## Data Availability

All data generated or analyzed during this study are included in this published article and its supplementary information files.

## Abbreviation

AI: Artificial intelligence

## Declarations

### Consortium

HealthTech Alliance

Jhancy Malay^1^, Sarah A. Haji^2^, Mawada A. H. Taha^3^, Fada S. Alzeidi^4^, Badr A. Al-Tayar^95^, Awlaa Adil^5^, Alaa Mahmoudy^6^, Hiba Y. Yaghmour^7^, Mariam A. Abdulhay^8^, Malak S. Mahdi^9^, Fatema Almadhoob^10^, Djehina Ferhat^11^, Amr Khaled^12^, Omaima Y. A. Al Shehhi^13^, Fatema I. J. Alshakhoori^10^, Eman S. N. Shubbar^15^, Hussam S. Aziz^9^, Younes F. Samara^17^, Noor Alhuda R. Mohammed^18^, Sharouq R. A. Al Rubaiei^19^, Tasnim Tarboush^20^, Mahmoud E. Kamal^21^, Fatma A. Al-shamsi^22^, Ahmad Al Othman^23^, Zohra Bensayah^24^, Danya Ibrahim^25^, Manal Al-Ghafri^26^, Amjd Sobh^27^, Butaina Abdulhafith^28^, Bayan S. M. J. A. Alawi^29^, Alshaima A. H. Koko^30^, Fatema M. Shehab^31^, Aaya E. Ahmed^32^, Fatema S. M. J. Alawi^33^, Shatha Almabrok^34^, Marwan Alqudah^35^, Fada M. H. Al-Ghfeeili^36^, Nagham Alkhateeb^37^, Mohammad H. Awde^38^, Dina E. Abozaid^39^, Taqi M. J. Taher^40^, Alaa Youness^41^, Mahmoud A. M. Albadawi^42^, Mohammad Darwish^43^, Rowayda Hamouda^44^, Mayameen H. Salman^45^, Kareem Ibraheem^46^, Lomass Soliman^47^, Noha Taymour^48^, Hanan A. S. Barbaid^49^, Haidar Kanso^50^, Hanan A. S. Bamatraf^51^, Khalil Smirat^52^, Afia Murshida^53^, Samar Al-Jabri^54^, Ahmed S. Al Sakini^55^, Wadea A. A. A. Abduh^56^, Ruqaia Shoheeduzzaman^57^, Fatima J. S. Bawazeer^58^, Haidy Samy^59^, Ali Alasassi^60^, Shawqi Seder^61^, Ahmad Alkheder^62^, Lina S. A. Alqumairi^63^, Ghadeer A. A. Al-Surabi^64^, Manel Ferjani^14^, Rania Essouei^16^, Ahmed G. Elmenady^65^, Nour Hamed^38^, Dalal J. Saed^66^, Amaal Dier^67^, Shima A. M. Musa^68^, Naris M. Kadoura^69^, Asma Maliha^70^, Juman Muhtaseb^71^, Rama A. Abu Doush^72^, Shahed M. Benghuzi^73^, Sara A. Araed^74^, Abdullah Khalailah^75^, Hind A. Abdulhay^76^, Omar I. Bsharat^77^, Rasha A. A. Salama^78^, Nour Moosa^79^, Baraa M. A. Khader^80^, Mirna F. Mustafa^81^, Husam Abu Dawood^82^, Ghalia Al Turki^83^, Amal Njoom^84^, Habib Alfalah^85^, Abdullah Ayad^86^, Sarah M. Abdelmohsen^87^, Ali Albashiri^88^, Amer O. Girnos^89^, Aya Kawssan^90^, Zoha Rasheed^91^, Rama Alsukhni^92^, Mahmoud N. Bani Hani^93^, Marwan F. Alzboon^95^, Muhammed M. Abdiwahab^96^, Solaima^97^, Nourin N.Sayed^98^, Zainab M. J. Malalla^99^, Abdullah M. Zahran^100^, Anas^101^, Wesam S. A. Elariny^102^, Alanoud AlShahwani^103^, Nooran A.Mohammed^104^

^1^Faculty of Pediatrics, RAK Medical and Health Sciences University, Ras Al Khaimah, United Arab Emirates

^2^Lecturer, Department of Oral and Maxillofacial Surgery, College of Dentistry, University of Basrah, Basrah, Iraq

^3^Surgery, National Ribat University, Faculty of Medicine, Khartoum, Sudan

^4^Student in the National University of Science and Technology, Sohar, Oman

^5^Faculty of Medicine, National University, Sohar, Sultanate of Oman

^6^Faculty of Science, Zagazig University, Department of Biochemistry, Giza

^7^Faculty of Medicine, Palestine Polytechnic University, Hebron, Palestine

^8^Faculty of Medicine, Mansoura–Manchester Program, Mansoura, Egypt

^9^Faculty of Medicine, Mansoura University, Mansoura, Egypt

^10^Faculty of Medicine, Alexandria University, Alexandria, Egypt

^11^Faculty of Medicine, Ben Youssef Ben Khadda University 1, Algiers, Algeria

^12^Faculty of Medicine, Assiut University, Assiut, Egypt

^13^College of Medicine, National University of Science and Technology

^14^Faculty of Dental Medicine, Monastir University, Monastir, Tunisia

^15^Faculty of Medicine, Alexandria University, Alexandria, Egypt

^16^IHEC Carthage, Carthage University, Tunis, Tunisia

^17^Hashimiyya University

^18^College of Dentistry, University of Basrah, Basrah 61004, Iraq

^19^National University of Science and Technology, College of Medicine and Health Sciences

^20^Faculty of Medicine, Mansoura University, Mansoura, Egypt

^21^Faculty of Medicine, Egyptian Russian University, Badr City, Egypt

^22^National University in Medicine, Muscat, Oman

^23^Faculty of Medicine, Near East University, Nicosia, North Cyprus

^24^Faculty of Medicine, Ben Youssef Ben Khadda University 1, Algiers, Algeria

^25^Faculty of Medicine, University of Khartoum, Khartoum, Sudan

^26^Final-year Medical Student, National University, Oman

^27^Faculty of Medicine, Tartous University, Tartous, Syria

^28^Faculty of Medicine, University of Tripoli, Tripoli, Libya

^29^Faculty of Medicine, Alexandria University, Alexandria, Egypt

^30^National Ribat University, Sudan

^31^Faculty of Medicine, Alexandria, Egypt

^32^Dubai Medical College for Girls, Dubai, United Arab Emirates

^33^Faculty of Medicine, Alexandria University, Alexandria, Egypt

^34^Faculty of Medicine, Mansoura University, Mansoura, Egypt

^35^Faculty of Medicine, Mansoura University, Mansoura, Egypt

^36^Medical Student, National University of Science and Technology, Sohar, Oman

^37^Faculty of Medicine, Palestine Polytechnic University, Hebron, Palestine

^38^Faculty of Medicine, Damascus University, Damascus, Syria

^39^Faculty of Dentistry, Tanta University, Tanta, Egypt

^40^Family and Community Medicine Department, College of Medicine, Wasit University, Wasit, Iraq

^41^Faculty of Medicine, Lebanese University, Al Hadath, Lebanon

^42^Faculty of Medicine, Kafrelsheikh University, Kafr El-Sheikh, Egypt

^43^Faculty of Medicine, Jordan University of Science and Technology

^44^Faculty of Medicine, Kafrelsheikh University, Kafr El-Sheikh, Egypt

^45^Graduated Medical Doctor, Wasit University

^46^Faculty of Medicine, Palestine Polytechnic University, Hebron, Palestine

^47^Institute of Pharmaceutical Technology and Regulatory Affairs, Faculty of Pharmacy, University of Szeged, Hungary

^48^Department of Substitutive Dental Sciences, College of Dentistry, Imam Abdulrahman Bin Faisal University, Dammam, Saudi Arabia

^49^Faculty of Medicine, Hadhramout University, Yemen

^50^Faculty of Medicine, Damascus University, Damascus, Syria

^51^Faculty of Medicine and Health Sciences, Hadhramout University, Yemen

^52^Faculty of Medicine, Jordan University of Science and Technology, Jordan

^53^Faculty of Health, School of Medicine and Population Health, University of Sheffield, UK

^54^College of Medicine and Health Sciences, National University of Science and Technology, Oman

^55^College of Medicine, University of Baghdad, Iraq

^56^University for Medical and Applied Sciences, Yemen

^57^Department of Biological and Environmental Sciences, Qatar University, Qatar

^58^Faculty of Medicine, Hadhramout University, Al-Mukalla, Yemen

^59^Faculty of Physical Therapy, Modern University for Technology and Information, Egypt

^60^Faculty of Clinical Pharmacy, Egyptian Russian University, Egypt

^61^Faculty of Medicine, Palestine Polytechnic University, Hebron, Palestine

^62^Faculty of Medicine, Damascus University & Syrian Private University, Damascus, Syria

^63^Graduate Doctor, National University, Oman

^64^Student, Medicine Department, Faculty of Medicine and Health Sciences, Sana’a University, Sana’a City, Yemen

^65^Faculty of Medicine, Mansoura University, Dakahlia, Egypt

^66^Faculty of Medicine, Tobruk University, Tobruk, Libya

^67^Faculty of Medicine, University of Tripoli, Libya

^68^Faculty of Medicine, Al-Neelain University, Khartoum, Sudan

^69^Mansoura Manchester Medical Program, Egypt

^70^Qatar University

^71^Faculty of Nursing, Palestine Polytechnic University, Hebron, Palestine

^72^Faculty of Dentistry, University of Jordan, Amman, Jordan

^73^Faculty of Medicine, Alexandria University, Alexandria, Egypt

^74^Faculty of Dentistry, University of Benghazi, Benghazi, Libya

^75^Faculty of Medicine, Alexandria university, Alexandria, Egypt

^76^Faculty of Medicine, Mansoura University – Manchester Program

^77^Faculty of Medicine, Kuwait

^78^Department of Community Medicine, Ras Al kHaimah Medical and Health Science University, Ras Al khaimah, UAE

^79^Faculty of Medicine, Palestine Polytechnic University, Hebron, Palestine

^80^Faculty of Medicine, Alexandria University, Egypt

^81^Faculty of Medicine, Al-Quds University, Palestine

^82^Faculty of Medicine, Palestine Polytechnic University, Hebron, Palestine

^83^Faculty of Medicine, Mansoura University Manchester Program

^84^Faculty of Medicine, Palestine Polytechnic University, Hebron, Palestine

^85^Faculty of Medicine, Jordan University of Science and Technology, Jordan

^86^College of Dentistry, University of Basrah, Basrah, Iraq

^87^Faculty of Medicine, Aswan University, Aswan, Egypt

^88^Faculty of Medicine, University of Science and Technology

^89^Faculty of Medicine, University of Duhok, Duhok, Iraq

^90^Faculty of Medicine, Lebanese University, Beirut, Lebanon

^91^University of Doha for Science and Technology

^92^Faculty of Medicine, Hashemite University, Al-Zarqa, Jordan

^93^Faculty of Medicine, Jordan University of Science and Technology (JUST)

^94^Faculty of Medicine and Health Sciences, University of Science and Technology, Sana’a, Yemen

^95^Faculty of Medicine, Alexandria University, Egypt

^96^Faculty of Medicine, Tanta University, Tanta, Egypt

^97^Affiliation not available

^98^Master’s in Psychology, University of Kerala, India

^99^Mansoura University, Egypt

^100^College of Medicine, Palestine Polytechnic University, Palestine

^101^Affiliation not available

^102^Faculty of Agriculture, Tanta University, Tanta, Egypt

^103^Hayat Universal School (HUBS), Qatar

^104^Faculty of Medicine, Ain Shams University, Cairo, Egypt
