# Supplementary material for: Public attitudes and practices toward using AI chatbots for healthcare assistance: a multinational cross-sectional study
Source: BMC Health Serv Res. 2025 Dec 30;26:335. doi: 10.1186/s12913-025-13832-0 (PMC12967010; doi:10.1186/s12913-025-13832-0)
Supplement: Supplementary file 1 — Supplementary Material 1 [file 12913_2025_13832_MOESM1_ESM.pdf]

Public attitudes and practices toward using AI chatbots for healthcare assistance: A Multinational Cross-Sectional Study

Authors:

Aya Elsayed Abdelwahed[1*], Mahmoud Abd El-Nasser[1], Omar Qasem Heih [2], Aya Muhammed Suleiman[1], Ahmed Mithqal Khader [2], Rahma AbdElfattah Ibrahim [3], Mohamed Rabiea Abdelnaby Fathalla Hamad[1], Eslam Radwan[1], Azza Magdy Srour [4], HealthTech Alliance, Marwa Mohammed Ibrahim Ghallab[5].

File content

- Table S1 & S2: Study Questionnaire
- Item coding
- Validity & reliability
- Table S3: Attitude Results Table (Original five-point scale)
- Table S4: Benjamini-Hochberg False Discovery Rate (FDR)
- Acknowledgement

Study questionnaire

Table S1 (English version):

<table>
<tr><td colspan="2">Assessing the perception and practices of the general population regarding the use of AI-powered chatbots in healthcare assistance in the Arab world.</td></tr>
<tr><td colspan="2"><u>Objectives</u>
This study aims to:
▪ Investigate the public awareness of AI chatbots and their uses in healthcare assistance.
▪ Assess the public attitude to use AI chatbots in healthcare assistance.
▪ Determine the public confidence regarding AI-powered chatbot use in healthcare.
▪ Estimate the prevalence of using AI chatbots for health care assistance.
▪ Correlate the demographic characteristics with knowledge, attitude, and practice of AI chatbots for health care assistance

Note: If you are less than 18 years old, ask parents to provide the consent to participate</td></tr>
<tr><td colspan="2">Filling out this questionnaire constitutes your informed consent to use your answers for research purposes only without revealing your identity or personal data and you are free to withdraw at any time, without giving a reason, thank you so much.</td></tr>
<tr><td colspan="2">Socio-demographic section</td></tr>
<tr><td>Age (Only numbers)</td><td></td></tr>
<tr><td>Gender</td><td>○ Gender
○ Female
○ Prefer not to answer</td></tr>
<tr><td>Marital Status</td><td>○ Married
○ Single
○ Widow
○ Divorced
○ Prefer not to answer</td></tr>
<tr><td>Country</td><td>○ Algeria
○ Bahrain
○ Comoros
○ Djibouti
○ Egypt
○ Iraq
○ Jordan</td></tr>
</table>

	o Kuwait<br>o Lebanon<br>o Libya<br>o Mauritania<br>o Morocco<br>o Oman<br>o Palestine<br>o Qatar<br>o Saudi Arabia<br>o Somalia<br>o Sudan<br>o Syria<br>o Tunisia<br>o United Arab Emirates<br>o Yemen
Residence	o Urban<br>o Rural
Education	o Non-educated.<br><br>o Pre-university education (including literacy programs, technical or vocational education, preparatory education, secondary education, etc.)<br><br>o University education (undergraduate/ postgraduate)<br><br>o Post-graduate degrees (Master's/Doctorate).
Main occupation	o Unemployed<br>o Governmental employee<br>o Private employee<br>o Freelancer<br>o Artisan (Examples of artisan jobs may include carpenter, blacksmith, jewelry maker, potter, or weaver.)<br>o Housewife<br>o Student<br>o Retired<br>o Other(please specify)
Your profession (current or future)	o Education<br>o Engineering<br>o Health care system<br>o IT/ computer science<br>o Business<br>o Arts / Humanities<br>o Natural science<br>o Other (please specify)
Diagnosed with chronic disease.	o Yes

(For example, (Diabetes, Cardiovascular diseases such as (High Blood Pressure, Heart Disease, Arrhythmia. Atrial tachycardia, High Blood Cholesterol), Gastrointestinal disease, Kidney disease, Liver disease, Respiratory system diseases)	○ No
Do you have any psychological or mental health issues?<br><br>(For example: (Anxiety disorders, Mood disorders, Psychotic disorders, eating disorders, Personality disorders, Obsessive-compulsive disorders, Trauma and stressor-related disorders, Substance use disorders, Sleep-wake disorders))	○ Yes<br>○ No
Hearing about AI chatbots	
Have you heard about AI-powered chatbots (like: Chat GPT, Gemini, Poe)<br><br>AI: Artificial intelligence	○ Yes (next question)<br>○ No (submit)
Based on the information you have, AI-powered chatbots are defined as	○ Chatbots that are powered by human intelligence.<br><br>○ Chatbots that are programmed to make phone calls.<br><br>○ Chatbots that rely on natural language processing and machine learning to mimic human conversation<br><br>○ Chatbots that are only used for audio
I have participated in training courses or workshops about AI-powered chatbots or similar content online.	○ Yes<br>○ No
Attitude toward using AI chatbots for health care assistance.	

**I think AI chatbots are effective to:**	I strongly disagree.	Disagree.	Neutral	Agree	strongly agree.
1. Contribute to health care assistance.					
2. Provide accurate and trustworthy sources of health-related information.					
3.Facilitate the accessibility of health-					

related information and resources					
4.Be used for identifying the initial symptoms assessment for health-related issues					
5.Assist in medication management and reminders.					
6. Offer psychological and mental support and resources.					
7. Contribute to remote monitoring of health issues					
8. Offer cost-effective solutions for healthcare assistance.					
9. Replace the human health care professionals.					
10. Help the human health care professionals.					
**Regarding using AI chatbots for health care assistance:**	I strongly disagree.	Disagree.	Neutral	Agree	strongly agree.
1. I feel comfortable when discussing health issues with AI chatbots (or imagining the situation if I haven't done it before).					
2. I consider it transparent.					
3. I consider it to be trustworthy.					

Practice

**Have you ever used AI chatbots for the following ((other than health care assistance)):**	Yes	No
Education and learning		
Scientific research		
Business		
Personal assistance		
Sales and marketing		
Banking and financial services		
Travel and hospitality.		
Other		

Have you ever used AI chatbots for healthcare assistance	○ Yes (next question)<br>○ No (submit)
Mention the chatbots you used.<br>(You can choose more than one)	☐ ChatGPT<br>☐ Gemini

	☐ Siri<br>☐ Alexa<br>☐ Google Assistant<br>☐ AI powered chatbots in some social media ((Snapchat, telegram, messenger…))<br>☐ Other (please specify)

You used AI chatbots in which of the following:	Yes	No
1. Medication management (and/ or) Reminders		
2. Assisting with appointment scheduling and reminders related to health (such as check-ups, medications, and any health regimen)		
3. Facilitating online health consultations		
4. Getting information about self-medication (Taking medicines without physician consultation)		
5. Offering personalized health coaching (such as promoting a healthy lifestyle with exercising, a healthy diet, quitting smoking, and more.)		
6. Online nursing and monitoring services		

Table S2 (Arabic version):

Note that the reliable Arabic version has an additional 4 options in the last question which were deleted from the English version after the pilot study as they reduced the reliability of this domain.

تقييم إدراك وممارسات عامة الناس لاستخدام برامج الدردشة الآلية المدعومة بالذكاء الاصطناعي في الرعاية الصحية في العالَم العربي
**الأهداف**<br>هذه الدراسة تهدف إلى:<br>▪ التحقق من معرفة العامة ببرامج الدردشة الآلية المدعومة بالذكاء الاصطناعي " AI powered chatbots" واستخداماتها فـ المساعدة في الرعاية الصحية.<br>▪ تقييم موقف العامة حول استخدام برامج الدردشة الآلية المدعومة بالذكاء الاصطناعي للمساعدة في الرعاية الصحية.<br>▪ تحديد ثقة العامة فيما يتعلق باستخدام برامج الدردشة الآلية المدعومة بالذكاء الاصطناعي في الرعاية الصحية

▪ معرفة مدى انتشار استخدام برامج الدردشة الآلية المدعومة بالذكاء الاصطناعي ف المساعدة في الرعاية الصحية.
▪ توصيف الخصائص السكانية وعلاقتها بالمعرفة والموقف والممارسة الخاصة باستخدام برامج الدردشة الآلية المدعومة بالذكاء الاصطناعي للمساعدة في الرعاية الصحية.

ملاحظة: إذا كان عمرك أقل من 18 عامًا، يرجى طلب موافقة والديك للمشاركة.

ملئ هذا الاستبيان يُعَد موافقة منك على استخدام إجاباتك لأغراض بحثية فقط بدون الكشف عن هويتك أو بياناتك الشخصية ولك كامل الحق في حرية الانسحاب في أي وقت بدون أسباب وشكراً جزيلاً.

الخصائص الاجتماعية والديموغرافية	
العمر (مسموح بأرقام فقط)	
الجنس	○ ذكر<br>○ أنثى<br>○ أفضل عدم الإجابة
الحالة الاجتماعية:	○ متزوج<br>○ أعزب<br>○ أرمل<br>○ مطلق<br>○ أفضل عدم الإجابة
الدولة	○ الجزائر<br>○ البحرين<br>○ جزر القمر<br>○ جيبوتي<br>○ مصر<br>○ العراق<br>○ الأردن<br>○ الكويت<br>○ لبنان<br>○ ليبيا<br>○ موريتانيا<br>○ المغرب<br>○ عمان<br>○ فلسطين<br>○ قطر<br>○ المملكة العربية السعودية<br>○ الصومال<br>○ السودان<br>○ سوريا<br>○ تونس<br>○ الإمارات العربية المتحدة<br>○ اليمن
الإقامة	○ الريف<br>○ الحضر
التعليم	○ غير مُتَعَلِّم<br>○ التعليم ما قبل الجامعي (بما في ذلك برامج محو الأمية والتعليم الفني أو المهني والتعليم الإعدادي / المتوسط والتعليم الثانوي، وما إلى ذلك)<br>○ التعليم الجامعي (البكالوريوس / خريج)

	○ الدراسات العليا (الماجستير / الدكتوراه)
الوضع المهني	○ لا أعمل<br>○ موظف حكومي<br>○ موظف في القطاع الخاص<br>○ عمل حرّ<br>○ حرفي (مثال على وظائف الحرفيين قد تشمل النجار والحداد وصانع المجوهرات والخزاف والنسّاج وما إلى ذلك)<br>○ ربة منزل<br>○ طالب<br>○ أخرى (من فضلك حدد)
التخصص الخاص بك (الحالي أو المستقبلي)	○ التعليم<br>○ الهندسة<br>○ الرعاية الصحية<br>○ تقنية المعلومات / علوم الحاسوب<br>○ الأعمال التجارية<br>○ آداب وعلوم إنسانية<br>○ العلوم الطبيعية<br>○ أخرى (من فضلك حدد)
تم تشخيصك بمرض مزمن من قبل (على سبيل المثال:ا لسكري أمراض القلب والأوعية الدموية مثل (ارتفاع ضغط الدم، أمراض القلب، اضطراب ضربات القلب، تسارع أذيني، ارتفاع الكوليسترول في الدم)،أمراض الجهاز الهضمي أمراض الكلى أمراض الكبد أمراض الجهاز التنفسي)	○ نعم<br>○ لا
هل تعاني من مشاكل خاصة بالصحة النفسية أو العقلية<br><br>على سبيل المثال: اضطرابات القلق،اضطرابات المزاج،الاضطرابات النفسية،أمراض الأكل،أمراض الشخصية،اضطرابات الاكتئاب المرضي،اضطرابات الصدمة والضغوط الناتجة عن الضغط،اضطرابات استخدام المخدرات ،اضطرابات النوم - الاستيقاظ	○ نعم<br>○ لا
**المعرفة عن بوت المحادثة الذي يعمل بالذكاء الاصطناعي**	
هل سمعت عن بوت المحادثة (الدردشة) الذي يعمل بالذكاء الاصطناعي؟<br>(مثل: Chat GPT، Gemini، Poe)	○ نعم (الانتقال الى الأسئلة التالية)<br>○ لا (تسليم الاستبيان)
بناءاً علي ما لديك من معلومات: يُعَرَّف بوت المحادثة (الدردشة) بالذكاء الاصطناعي على أنه	○ بوت محادثة (دردشة) مدعوم بالذكاء البشري.

	○ بوت محادثة (دردشة) مُبرمج لإجراء مكالمات هاتفية.<br>○ بوت محادثة (دردشة) يعتمد على معالجة اللغة الطبيعية والتعلم الآلي لتقليد المحادثة البشرية.<br>○ بوت محادثة (دردشة) مستخدم للصوت فقط.
لقد شاركت في دورات تدريبية أو ورش عمل عن الروبوتات المحادثة المدعومة بالذكاء الاصطناعي، أو محتوى مماثل عبر الإنترنت	○ نعم<br>○ لا

الموقف والإدراك

**أعتقد أن بوت الدردشة بالذكاء الاصطناعي فعال في:**	لا أوافق بشدة.	لا أوافق.	حيادي	أوافق	أوافق بشدة.
1.تقديم المساعدة في مجال الرعاية الصحية.					
2.توفير مصادر دقيقة وموثوقة للمعلومات المتعلقة بالصحة					
3.تسهيل إمكانية الوصول إلى المعلومات والمصادر المتعلقة بالصحة					
4.يمكن استخدامها لمعرفة الأعراض الأولية للمشاكل الصحية					
5.المساعدة في تنظيم وتذكير بمواعيد الأدوية					
6.تقديم مصادر الدعم الخاص بالصحة النفسية والعقلية					
7.المساهمة في تقييم ومتابعة الحالة الصحية					
8.تقديم الحلول الصحية بتكلفة مناسبة وفعّالة.					
9.تستبدل الطاقم الطبي لتقديم الرعاية الصحية					
10.أن تساعد في إكمال عمل الطاقم الطبي لتقديم الرعاية الصحية					
**فيما يتعلق باستخدام بوت الدردشة بالذكاء الاصطناعي للمساعدة في الرعاية الصحية :**	لا أوافق بشدة.	لا أوافق.	حيادي	أوافق	أوافق بشدة.
1.أشعر بالارتياح عند مناقشة المشكلات الصحية مع بوت الدردشة بالذكاء الاصطناعي (أو عند تخيلك للموقف في حالة أنك لم تفعل من قبل)					
2.أعتبرها مصدر ذا شفافية.					
3.أعتبرها مصدر موثوق فيه.					

الممارسة

هل سبق لك استخدام بوت الدردشة المدعوم بالذكاء الاصطناعي للأغراض التالية (غير الرعاية الصحية)؟	نعم	لا

التعليم والتعلم		
البحث العلمي		
العمل (المهنة)		
المساعدة الشخصية		
المبيعات والتسويق		
الخدمات المصرفية والمالية		
السفر والضيافة (الخدمات السياحية مثل الفنادق والمطاعم وشركات السفر والجولات السياحية وغيرها)		
أخرى		

هل سبق لك استخدام بوت الدردشة بالذكاء الاصطناعي للمساعدة في الرعاية الصحية	○ نعم (الانتقال للأسئلة التالية)<br>○ لا (تسليم الاستبيان)
اختر بوت المحادثة الذي استخدمته (يمكنك اختيار أكثر من اختيار)	□ ChatGPT<br>□ Gemini<br>□ Siri<br>□ Alexa<br>□ Google Assistant<br>□ AI powered chatbots in some social media. ((snapchat, telegram, messenger…))<br>□ أخرى (يرجى التحديد)

لقد استخدمت بوت الدردشة بالذكاء الاصطناعي في اى مما يلي:	نعم	لا
1.التذكير بمواعيد الأدوية		
المساعدة في تنظيم والتذكير بالمواعيد المتعلقة بالأمور الصحية (مثل مواعيد الكشف الطبي وتناول الأدوية، وأي أنظمة صحية أخرى)		
3.تسهيل الاستشارات الطبية عبر الإنترنت		
4.الحصول على معلومات حول العلاج الذاتي (تناول أدوية بدون استشارة الطبيب)		
5.المساعدة في إرشادات صحية شخصية ((نمط الحياة الصحي مثل ممارسة التمارين الرياضية ونظام غذائي صحي وتوقف التدخين وغير ذلك))		
6.تقديم خدمات التمريض والمتابعة عبر الإنترنت		
7.تقييم وفحص الأعراض الأولية للأمراض		
8.التشخيص الذاتي		
9.الدعم المتعلق بالصحة النفسية والعقلية		
10.أخرى		

Item coding

Demographic data:

- Gender coded 0 for “prefer not answering”, 1 for “female”, and 2 for “male”.

- Marital status coded 0 for “prefer not answering”, 1 for “divorced”, 2 for “widow”, 3 for “single”, and 4 for “married”.

- Residency coded 1 for “urban”, and 2 for “rural”.

- Education is coded 0 for “uneducated”, 1 for “preuniversity education”, 2 for “university education”, and 3 for “postgraduate degree”.

- Main occupation and (main specialty) were coded from 1 for “unemployed” (“education”) to 8 for “other occupation” (“other specialty”).

- Both chronic illness and psychological illness are coded 1 for “no”, and 2 for “yes”.

Knowledge about AI Chatbots:

- Hearing about AI chatbots (participants who had not heard about AI chatbots submitted the questionnaire after this question).

- Definition of AI chatbots: “Chatbots that rely on natural language processing and machine learning to mimic human conversation" is coded 1, and other answers are coded 0.

- Previous training in AI was coded 1 for “no”, and 2 for “yes”.

Attitude:

The participants were asked to rate each option on a five-point Likert scale, with 1 indicating "Strongly Disagree," and 5 indicating "Strongly Agree."

We changed the original 5-point scale to a simpler 3-point scale for easier interpretation. "Strongly Disagree" and "Disagree" were combined and labeled as "Disagree". The "Neutral" option remains unchanged, while "Agree" and "Strongly Agree" were merged into a single category labeled "Agree".

Practice of AI Chatbots in Healthcare Assistance:

Each option is coded 1 for “no” or 2 for “yes”.

Validity and reliability

We validated and refined the questionnaire through a multi-step process.

1. Content validity details

Content Validity was ensured by six public health and epidemiology experts who assessed relevance, clarity, and comprehensiveness. The item and scale Content validity index was calculated to be as the following:

Statement: "The AI-powered chatbots are defined as"

- I-CVI: range from ≈ 0.83 to 1 for Clarity, comprehensiveness, and relevance

---

Statement: "I have participated in training courses or workshops about AI-powered chatbots or similar content online."

- I-CVI: range from ≈ 0.83 to 1 for Clarity, comprehensiveness, and relevance

---

Third Domain: Attitude Questions

- Range of I-CVI: ≈ 0.83 to 1 for clarity, comprehensiveness, and relevance
- Scale Details:
  - For 5 items: All experts rated 3 or 4 → I-CVI = 1.00
  - For 5 items: 1 expert rated 2, others rated 3 or 4 → I-CVI = 0.83
- S-CVI/Ave: 0.915 for clarity, comprehensiveness, and relevance

---

Fourth Domain: Confidence Questions

- Total items = 6
  - 4 items: All experts rated 3 or 4 → I-CVI = 6/6 = 1.00
  - 2 items: 5 experts rated 3 or 4, 1 expert rated 2 → I-CVI = 5/6 ≈ 0.83
- Item scores: ≈ 0.83 to 1 for clarity, comprehensiveness, and relevance
- S-CVI/Ave: 0.943

After the pilot test we removed 3 items of this domain with lower reliability including the 2 items rated 2 for clarity, comprehensiveness, and relevance in this scale, so the item CVI and Scale CVI is considered 1 in the final version of the questionnaire.

Fifth Domain: Practice

- Total items = 10

    - 6 items: All experts rated 3 or 4 → I-CVI = 6/6 = 1.00
    - 4 items: 5 experts rated 3 or 4, 1 expert rated 2 → I-CVI = 5/6 ≈ 0.83
- Item scores: ≈ 0.83 to 1 for clarity, comprehensiveness, and relevance
- S-CVI/Ave:

S-CVI/Ave=0.932

Construct Validity

Construct validity was assessed through Exploratory Factor Analysis (EFA) using Principal Axis Factoring to identify latent structures and Oblimin rotation for expected factor correlations, yielding a KMO ranging from 0.65 to 0.89 and a significant Bartlett's test ($p < 0.001$), confirming sample adequacy. Items with factory loads above 0.40 were retained.

Confirmatory Factor Analysis (CFA) confirmed the validity of our questionnaire, demonstrating a satisfactory factor structure with strong factor loadings for most items. Model fit indices were assessed, and necessary modifications were made to enhance the model's robustness. For the refined models (after removing problematic items), the Root Mean Square Error of Approximation (RMSEA) values ranged from 0.000 to 0.131, reflecting improved model fit. The Comparative Fit Index (CFI) ranged from 0.753 to 1.000, and the Tucker-Lewis Index (TLI) ranged from 0.692 to 1.000, indicating moderate to excellent fit across domains. The Standardized Root Mean Square Residual (SRMR) values varied between approximately 0 and 0.0826, with most values falling within the acceptable threshold (<0.08), suggesting generally good model fit after item refinement.

English version

Exploratory Factor Analysis – Attitude Domain (English)

Item	Factor 1	Factor 2	Uniqueness
1. Contribute to health care assistance	0.783		0.409
2. Provide accurate and trustworthy sources of health-related information	0.572		0.476
3. Facilitate the accessibility of health-related information and resources	0.848		0.307
4. Identify initial symptoms assessment for health-related issues	0.726		0.426
5. Assist in medication management and reminders	0.717		0.503
6. Offer psychological and mental support and resources	0.590		0.552
7. Contribute to remote monitoring for health issues	0.688		0.443
8. Offer cost-effective solutions for healthcare assistance	0.720		0.422

9. Replace human health care professionals		0.728	0.472
10. Help human health care professionals	0.718		0.502

Exploratory Factor Analysis – Confidence Domain (Before Removing Items)

Item	Factor 1	Factor 2	Uniqueness
1. I feel comfortable when discussing health issues with AI chatbots.	0.795		0.361
2. I consider it transparent.	0.732		0.459
3. I consider it trustworthy.	0.726		0.475
4. I find it strange / not familiar		0.861	
5. It can be misused for health assistance		0.999	0.001

Exploratory Factor Analysis – Confidence Domain (After Removing Items)

Item	Factor 1	Uniqueness
1. I feel comfortable when discussing health issues with AI chatbots.	0.765	0.414
2. I consider it transparent.	0.750	0.438
3. I consider it trustworthy.	0.734	0.461

Confirmatory Factor Analysis – Attitude Domain (English)

Item	Estimate	SE	Lower CI	Upper CI	Z	p
1. Contribute to health care assistance	0.886	0.0983	0.694	1.079	9.02	<.001

2. Provide accurate and trustworthy information	0.780	0.1023	0.580	0.981	7.63	<.001
3. Facilitate accessibility of health information	1.021	0.1015	0.822	1.220	10.06	<.001
4. Identify initial symptoms	1.005	0.1094	0.790	1.220	9.18	<.001
5. Assist in medication management	0.824	0.1087	0.611	1.037	7.58	<.001
6. Offer psychological support	0.814	0.1089	0.601	1.028	7.48	<.001
7. Remote monitoring	0.875	0.1017	0.676	1.074	8.60	<.001
8. Cost-effective solutions	0.899	0.0980	0.707	1.091	9.18	<.001
9. Replace human professionals	-0.167	0.0963	-0.356	0.021	-1.74	0.082
10. Help human professionals	0.826	0.1024	0.625	1.027	8.07	<.001

Confirmatory Factor Analysis – Confidence Domain (Before Removing Items)

Item	Estimate	SE	Lower CI	Upper CI	Z	p
1. Comfortable discussing health issues	0.937	0.112	0.718	1.156	8.38	<.001
2. Transparent	0.776	0.097	0.585	0.966	7.98	<.001
3. Trustworthy	0.791	0.102	0.592	0.990	7.79	<.001
4. Strange / unfamiliar	0.250	0.115	0.024	0.475	2.17	0.030
5. Can be misused	-0.084	0.130	-0.338	0.171	-0.64	0.520

Confirmatory Factor Analysis – Confidence Domain (After Removing Items)

Item	Estimate	SE	Lower CI	Upper CI	Z	p
1. Comfortable discussing health issues	0.916	0.111	0.698	1.133	8.24	<.001
2. Transparent	0.785	0.097	0.594	0.975	8.07	<.001
3. Trustworthy	0.802	0.101	0.603	1.000	7.90	<.001

Confirmatory Factor Analysis – Practice Domain (Before Removing Items)

Item	Estimate	SE	Lower CI	Upper CI	Z	p
1. Medication management and/or reminders	0.239	0.075	0.092	0.385	3.20	0.001
2. Appointment scheduling & reminders	0.208	0.082	0.047	0.368	2.54	0.011
3. Online health consultations	0.323	0.071	0.184	0.463	4.55	<.001
4. Info about self-medication	0.176	0.085	0.009	0.344	2.07	0.039
5. Personalized health coaching	0.174	0.079	0.020	0.329	2.21	0.027
6. Online nursing/monitoring	0.328	0.052	0.226	0.431	6.29	<.001
7. Identifying symptoms	0.106	0.079	-0.049	0.260	1.34	0.180
8. Self-diagnosis	-0.226	0.077	-0.377	-0.075	-2.94	0.003
9. Psychological & mental support	0.157	0.066	0.028	0.286	2.38	0.017
10. Others	0.187	0.085	0.021	0.353	2.21	0.027

Confirmatory Factor Analysis – Practice Domain (After Removing Items)

Item	Estimate	SE	Lower CI	Upper CI	Z	p

1. Medication management and/or reminders	0.272	0.079	0.118	0.427	3.45	<.001
2. Appointment scheduling & reminders	0.297	0.078	0.145	0.449	3.84	<.001
3. Online health consultations	0.381	0.070	0.243	0.519	5.42	<.001
4. Info about self-medication	0.206	0.081	0.048	0.364	2.55	0.011
5. Personalized health coaching	0.118	0.084	-0.047	0.283	1.40	0.161
6. Online nursing/monitoring	0.267	0.054	0.162	0.373	4.96	<.001

Arabic version

Exploratory Factor Analysis – Attitude Domain (Arabic - Translated)

Item	Factor 1	Factor 2	Uniqueness
1. Providing health care assistance	0.571		0.527
2. Providing accurate and reliable health information sources	0.428		0.623
3. Facilitating access to health information and resources	0.805		0.425
4. Identifying initial symptoms of health issues	0.507		0.561
5. Assisting in medication reminders	0.834		
6. Providing mental health support and resources	0.405		0.522
7. Contributing to health status evaluation and monitoring	0.670		0.369

8. Offering cost-effective healthcare solutions	0.645		0.434
9. Replacing the medical team in healthcare provision		-0.789	0.495
10. Supporting medical team in healthcare provision	0.438		0.634

Exploratory Factor Analysis – Confidence Domain (Arabic - Translated)

Item	Factor 1	Factor 2	Uniqueness
1. I feel comfortable discussing health issues with the chatbot	0.638		0.591
2. I consider it transparent	0.842		0.293
3. I consider it trustworthy	0.670		0.544
4. I find it strange / unfamiliar		0.999	0.003
5. It may be misused in health assistance		0.887	

Exploratory Factor Analysis – Confidence Domain (Arabic - Translated, After Removing Items)

Item	Factor 1	Uniqueness
1. I feel comfortable discussing health issues with the chatbot	0.635	0.597
2. I consider it transparent	0.842	0.290

3. I consider it trustworthy	0.672	0.548

Confirmatory Factor Analysis – Practice Domain (Arabic - Translated)

Item	Estimate	SE	Lower CI	Upper CI	Z	p
1. Participated in training or workshops on AI chatbots	0.036	0.026	-0.015	0.087	1.38	0.167
2. Used chatbot for medication reminders	0.144	0.045	0.057	0.231	3.23	0.001
3. Appointment scheduling and health-related reminders	0.167	0.047	0.074	0.260	3.51	<.001
4. Facilitating online medical consultations	0.285	0.046	0.196	0.375	6.24	<.001
5. Getting information about self-medication	0.335	0.043	0.249	0.420	7.71	<.001
6. Personalized health guidance (healthy lifestyle, diet, etc.)	0.241	0.041	0.161	0.321	5.92	<.001
7. Nursing and	0.305	0.044	0.219	0.392	6.93	<.001

monitoring services online						
8. Identifying initial symptoms of diseases	0.381	0.042	0.298	0.463	9.04	<.001
9. Self-diagnosis	-0.329	0.044	-0.416	-0.243	-7.46	<.001
10. Mental and psychological support	0.317	0.045	0.229	0.405	7.07	<.001
11. Other uses	0.168	0.048	0.074	0.261	3.51	<.001

Confirmatory Factor Analysis – Attitude Domain (Arabic - Translated)

Item	Estimate	SE	Lower CI	Upper CI	Z	p
1. Providing healthcare assistance	0.631	0.049	0.535	0.728	12.78	<.001
2. Providing accurate and reliable health information	0.649	0.055	0.542	0.756	11.90	<.001
3. Facilitating access to health info/resources	0.504	0.049	0.409	0.600	10.36	<.001
4. Identifying initial symptoms	0.639	0.049	0.543	0.735	13.04	<.001
5. Assisting with	0.421	0.057	0.310	0.533	7.39	<.001

medication scheduling						
6. Providing mental and psychological support	0.750	0.052	0.648	0.852	14.41	<.001
7. Monitoring health status	0.803	0.050	0.706	0.901	16.14	<.001
8. Offering cost-effective healthcare	0.790	0.053	0.687	0.894	14.96	<.001
9. Replacing healthcare professionals	-0.588	0.063	-0.712	-0.464	-9.32	<.001
10. Assisting healthcare professionals	0.716	0.060	0.598	0.835	11.86	<.001

Confirmatory Factor Analysis – Confidence Domain (Arabic - Translated, Before Removing Items)

Item	Estimate	SE	Lower CI	Upper CI	Z	p
1. Comfortable discussing health issues	0.675	0.058	0.560	0.789	11.59	<.001
2. Transparent	0.848	0.058	0.735	0.961	14.71	<.001
3. Trustworthy	0.692	0.057	0.581	0.803	12.20	<.001
4. Strange / unfamiliar	0.031	0.061	-0.089	0.151	0.51	0.611
5. Can be misused	-0.022	0.063	-0.145	0.101	-0.34	0.731

Confirmatory Factor Analysis – Confidence Domain (Arabic - Translated, After Removing Items)

Item	Estimate	SE	Lower CI	Upper CI	Z	p
1. Comfortable discussing health issues	0.671	0.058	0.557	0.785	11.5	<.001
2. Transparent	0.853	0.058	0.741	0.966	14.8	<.001
3. Trustworthy	0.689	0.057	0.578	0.800	12.2	<.001

Assumption Checks – Bartlett's Test

Domain	Test	χ^2	df	p
Attitude (EFA)	Bartlett's Test	540	45	<.001
Attitude (Arabic)	Bartlett's Test	1220	45	<.001
Confidence (EFA)	Bartlett's Test	122	10	<.001
Confidence (Arabic)	Bartlett's Test	327	10	<.001
Confidence (After Item Removal)	Bartlett's Test	99.1	3	<.001

Kaiser-Meyer-Olkin (KMO) Measure of Sampling Adequacy

Domain	Overall KMO
Attitude	0.883
Attitude (Arabic)	0.890
Confidence	0.651
Confidence (Arabic)	0.613

Confidence (After Item Removal)	0.709

Model Fit Indices – Confirmatory Factor Analysis

	Domain	χ²	df	p	RMSEA	CFI	TLI	SRMR
English	Attitude	83.2	35	<.001	0.110	0.908	0.881	0.0625
	Confidence (Before removing)	18.4	5	0.002	0.154	0.884	0.767	0.0740
	Confidence (After removing)	≈0	0	NaN	0.000	1.000	1.000	≈0
	Practice (Before removing)	77.9	35	<.001	0.167	0.581	0.461	0.127
	Practice (After removing)	15.8	9	0.072	0.131	0.875	0.791	0.0702
Arabic	Attitude	160	35	<.001	0.0997	0.895	0.865	0.0553
	Confidence (Before Removing)	57.2	5	<.001	0.170	0.837	0.674	0.0796
	Confidence (After removing)	≈0	0	NaN	0.000	1.000	1.000	≈0
	Practice	130	44	<.001	0.0736	0.753	0.692	0.0826

Reliability

	Cronbach's α	
**Domain**	**Arabic**	**English**
**Attitude**	0.76	0.87
**Confidence**	0.756	0.792
**practice**	0.795 (without removing items)	0.74 (after removing the last 4 items)
**Total**	0.874	0.85

Table S3: The attitude toward using AI chatbots for healthcare assistance (merged version for simplification)

			Frequency (n=9337)	Percent %
I think AI-powered chatbots are effective to*:	Contribute to health care assistance·	Disagree	2099	22·5
		Neutral	2755	29·5
		Agree	4483	48·0
		Disagree	2632	28·2
		Neutral	3170	34·0

	Provide accurate and trustworthy sources of health-related information·	Agree	3535	37·9
	Facilitate the accessibility of health-related information and resources	Disagree	1095	11·7
		Neutral	1955	20·9
		Agree	6287	67·3
	Be used for identifying the assessment of the initial symptoms for health-related issues	Disagree	1966	21·1
		Neutral	2555	27·4
		Agree	4816	51·6
	Assist in medication management and reminders·	Disagree	1471	15·8
		Neutral	2012	21·5
		Agree	5854	62·7
	Offer psychological and mental support and resources·	Disagree	2587	27·7
		Neutral	2993	32·1
		Agree	3757	40·2
	Contribute to remote monitoring of health issues	Disagree	2952	31·6
		Neutral	2905	31·1
		Agree	3480	37·3
	Offer cost-effective solutions for healthcare assistance·	Disagree	3076	32·9
		Neutral	2921	31·3
		Agree	3340	35·8
	Replace the human health care professionals·	Disagree	6747	72·3
		Neutral	1389	14·9
		Agree	1201	12·9
	Help human health care professionals·	Disagree	2853	30·6
		Neutral	2548	27·3

		Agree	3936	42·2
Regarding using AI-powered chatbots for healthcare assistance*:	I feel comfortable when discussing health issues with AI chatbots (or imagining the situation if I haven't done it before)	Disagree	3603	38·6
		Neutral	3047	32·6
		Agree	2687	28·8
	I consider it transparent·	Disagree	2890	22·5
		Neutral	3569	29·5
		Agree	2878	48·0
	I consider it to be trustworthy ·	Disagree	4163	28·2
		Neutral	3245	34·0
		Agree	1929	37·9

*Here, we changed the original 5-point scale to a simpler 3-point scale for easier interpretation. "Strongly Disagree" and "Disagree" were combined and labeled as "Disagree." The "Neutral" option remained unchanged, while "Agree" and "Strongly Agree" were merged into a single category labeled "Agree".

Table S4: Benjamini-Hochberg False Discovery Rate (FDR)

Variable	Raw p-value	Rank (i)	B-H Critical Value	Significant after FDR?
Age & Chatbot Usage	< 0.001	1	0.005	☑ Yes
Gender & Chatbot Usage	< 0.001	2	0.01	☑ Yes
Marital Status & Chatbot Usage	< 0.001	3	0.015	☑ Yes
Education & Chatbot Usage	< 0.001	4	0.02	☑ Yes
Occupation & Chatbot Usage	< 0.001	5	0.025	☑ Yes
Specialty & Chatbot Usage	< 0.001	6	0.03	☑ Yes
Mental Health Issues & Chatbot Usage	< 0.001	7	0.035	☑ Yes
AI Training & Chatbot Usage	< 0.001	8	0.04	☑ Yes
Residence & Chatbot Usage	0.326	9	0.045	✗ No

Chronic Disease & Chatbot Usage	0.619	10	0.05	✖ No
